# Biogeographic history of the Late Pleistocene and Holocene European small hamsters (subfamily Cricetinae)

**DOI:** 10.1038/s41598-025-34298-4

**Published:** 2026-01-08

**Authors:** Barbara Bujalska, Michał Golubiński, Danijela Popović, Claudio Berto, Nicholas J. Conard, Anna Lemanik, Elisa Luzi, Zoran Marković, Adam Nadachowski, Vasil Popov, Ivan Horáček, Mateusz Baca

**Affiliations:** 1 Centre of New Technologies, University of Warsaw, Warsaw, Poland; 2 Faculty of Archaeology, University of Warsaw, Warsaw, Poland; 3 Department of Early Prehistory and Quaternary Ecology, University of Tübingen, Tübingen, Germany; 4 Institute of Systematics and Evolution of Animals, Polish Academy of Sciences, Kraków, Poland; 5 Institue for Archaeological Sciences, University of Tübingen, Tübingen, Germany; 6 Natural History Museum, Belgrade, Serbia; 7 Institute of Biodiversity and Ecosystem Research, Bulgarian Academy of Sciences, Sophia, Bulgaria; 8 Department of Zoology, Charles University, Prague, Czechia

**Keywords:** Late Pleistocene, Cricetinae, Ancient DNA, Recolonisation, Hamster, Ecology, Ecology, Evolution, Zoology

## Abstract

The prevailing paleobiogeographic hypothesis suggests that many steppe and tundra-steppe taxa currently found in Central Asia expanded into Europe during Pleistocene glacial periods, when open habitats dominated. However, previous studies have shown that one such species, the European narrow-headed vole (*Stenocranius anglicus*), diverged from its Asiatic counterparts over 200 thousand years ago, implying its prolonged isolation and survival in European refugia through the Eemian interglacial period. To test whether this was an exception or part of a broader pattern, we analysed the mitochondrial genomes from 33 Late Pleistocene and Holocene small hamster (Cricetinae) remains from Central and Western Europe, the Balkans, and Anatolia, all previously identified morphologically as grey dwarf hamster (*Nothocricetulus migratorius*). Contrary to expectations, 16 Late Pleistocene Central European samples were assigned to the hairy-footed hamster (*Cricetiscus sungorus*), a species currently restricted to northern Kazakhstan and southern Russia, whereas 17 samples from the Balkans and Anatolia belonged to the grey dwarf hamster. In both cases, the Late Pleistocene samples formed clades sister to modern populations; however, with relatively recent divergence times. This suggests population continuity or repeated expansion of hamsters from Asiatic sources rather than long-term isolation in Europe, as previously observed in narrow-headed voles. Our findings indicate that steppe species, despite occupying similar ecological niches, respond to past climate change in a species-specific manner. Therefore, broad generalisations regarding their evolutionary history may be misleading.

**Supplementary Information:**

The online version contains supplementary material available at 10.1038/s41598-025-34298-4.

## Introduction

During the Late Pleistocene, many mammalian species currently limited to Asia expanded their range into Europe. This includes both large species, such as the saiga antelope (*Saiga tatarica*)^1,2^ and dhole (*Cuon alpinus*)^3^, and small species, such as the Kyrgyz grey vole (*Microtus ilaeus*)^4^, steppe lemming (*Lagurus lagurus*)^5^, steppe pika (*Ochotona pusilla*)^6^, collared lemming (*Dicrostonyx torquatus*)^7^, great jerboa (*Allactaga major*)^5^, and hamsters of the subfamily Cricetinae^8^*.* The detailed biogeographic histories of most of these species are not known, but the prevailing hypothesis posits that they expanded into Europe during colder and more arid periods when tundra and steppe-tundra environments dominated the continent and retreated to the east during warmer phases when steppe environments were replaced by forests^5,9^.

One of the species that is supposed to follow this model is the narrow-headed vole (*Stenocranius gregalis*), which currently inhabits the Central Asian steppes and parts of the northern Siberian tundra. However, during the Late Pleistocene glaciation, it was the dominant element of rodent communities across Central and Western Europe. Recently, an investigation of mitochondrial DNA from Late Pleistocene European narrow-headed voles revealed that they diverged from the Asiatic populations more than 200 thousand years (ka) ago and represent a distinct species, *Stenocranius anglicus*^10,11^. The extensive genetic record covering nearly the last 100 ka showed strict partitioning of mitochondrial lineages into Europe and Asia, suggesting prolonged isolation of these populations and survival of the European population, at least the Eemian Interglacial in some refugial areas in Europe^11^. The latter clearly deviates from the contraction-expansion model, suggesting that the Late Pleistocene biogeographic histories of steppe species may have been more complex and diversified but this has yet to be explored in other ecologically similar species such as the Asiatic small hamsters.

Small hamsters encompassing the genera *Nothocricetulus*, *Allocricetus*, and *Cricetiscus* (formerly *Phodopus*) are now limited to the Central Asian and Eastern European steppe belt, similar to most narrow-headed vole populations^12^. In the Pleistocene, the remains of small hamsters were recorded as an infrequent but constant element in European small mammal assemblages^8^. Traditionally, Early and Middle Pleistocene hamster remains were identified as *Allocricetus bursae* Schaub, 1930^13–15^, while the Late Pleistocene remains were assigned to the recent *N. migratorius*^8,16,17^*.* The Early and Middle Pleistocene records seem generally larger in size than the Late Pleistocene records, with the boundary on the Eemian Interglacial suggesting a different origin of the two groups, consistent with the expansion-contraction model^8^.

To assess whether the evolutionary history of narrow-headed voles represents an exception or reflects a broader pattern among steppe taxa, we examined mitochondrial DNA from Late Pleistocene small hamster remains recovered from various European palaeontological sites.

## Results

**Fig. 1:**
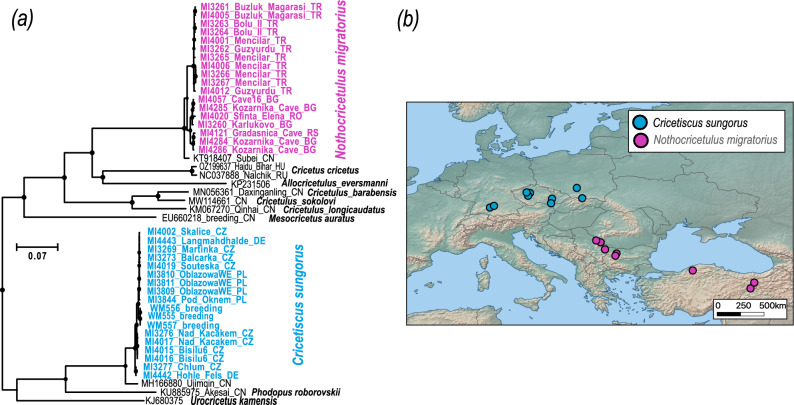
(**a**) Mitogenomic phylogeny of Cricetinae. Tips are labelled with lab numbers, palaeontological sites, and country codes. Sequences from GenBank are annotated with accession numbers, locality names, and country codes. For some sequences, the sampling location was not available. Black dots at nodes indicate ultrafast bootstrap support >0.95. (**b**) Map showing the sampling sites of ancient specimens and their taxonomic assignment. The base map was downloaded from Natural Earth @naturalearthdata.com and modified using QGIS @qgis.org.

We reconstructed partial mitogenomes from 33 Late Pleistocene and Holocene small hamster remains originating from 20 palaeontological sites across Central and Eastern Europe, as well as from three modern hairy-footed hamsters (*C. sungorus*). The mean coverage of the assembled mitogenomes ranged from 3× to 1076× (median: 144×), and the fraction of recovered mitogenome sequences varied between 0.5 and 1 (median: 0.88) (Figure 1; Supplementary Table S1). All ancient samples exhibited an increase in deamination at the ends of the DNA molecules (Supplementary Figures S1 and S2), a pattern characteristic of ancient DNA.

To determine the taxonomic position of the studied specimens, we reconstructed a phylogeny of Cricetidae (Figure 1a) using the available mitochondrial genomes. The resulting topology matched previous phylogenies based on mtDNA cytochrome *b* or mtDNA genomes^18–20^. The Late Pleistocene samples from Europe were grouped with two species: the grey dwarf hamster (*N. migratorius*) and the hairy-footed hamster (*C. sungorus*). The distribution of these two species seems non-random, as samples identified as grey dwarf hamsters originated only from sites in the Balkans, whereas samples identified as hairy-footed hamsters originated only from sites in Central and Western Europe (Figure 1b).

To further investigate the relationships between European Late Pleistocene and modern Asiatic populations, we used BEAST 1.10.5 software to reconstruct calibrated phylogenies for each hamster species separately.

**Fig. 2:**
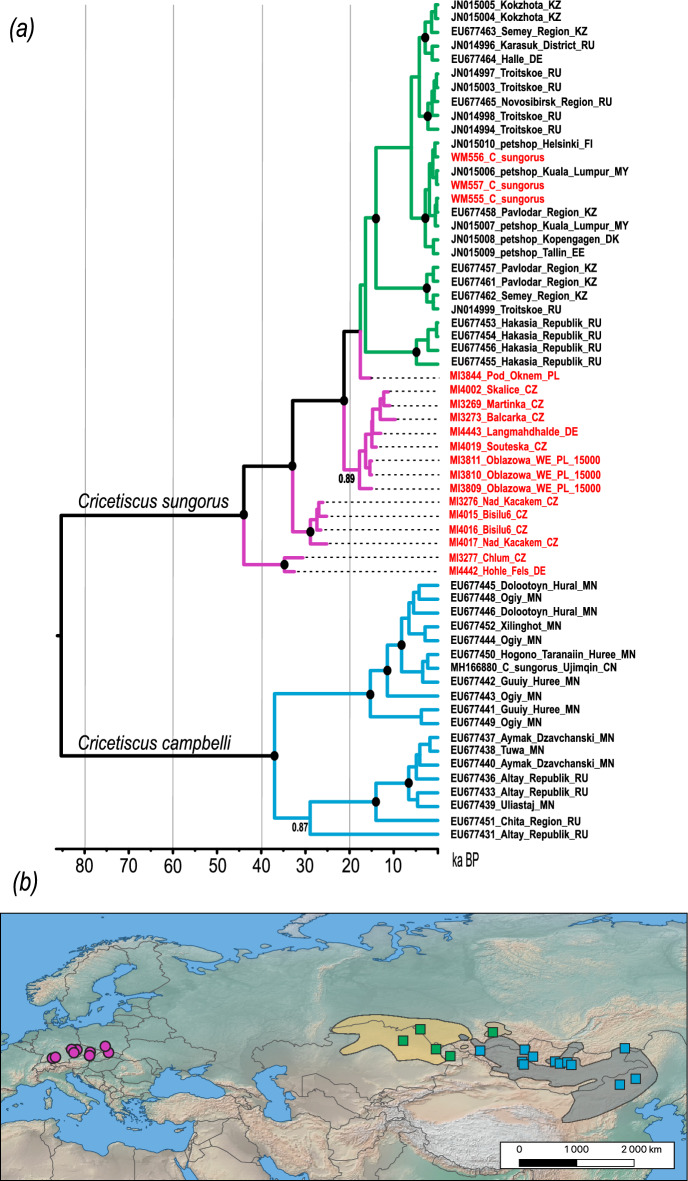
(**a**) Calibrated phylogeny of hairy-footed hamsters (*Cricetiscus* sp.). Black dots at nodes indicate posterior probability >0.9. Tips are annotated with laboratory IDs, palaeontological sites, and country codes. Tips of sequences from GenBank are annotated with accession numbers, sampling localities, and country codes. (**b**) Map showing the sampling locations of ancient and modern specimens. Circles represent samples newly sequenced in this study, while squares represent samples from GenBank. The yellow shaded area indicates the current distribution of *C. sungorus*, and the grey shaded area represents the distribution of *C. campbelli*, according to the IUCN. The base map was downloaded from Natural Earth @naturalearthdata.com and modified using QGIS @qgis.org.

The hairy-footed hamster (*C. sungorus*) dataset comprised 2230 bp long mtDNA sequences encompassing cytochrome *b*, control region, tRNA-Thr, and tRNA-Pro and included sequences of modern *C. sungorus* (n=25), *Cricetiscus campbelli* (n=18) selected from GenBank, and all our ancient and modern *C. sungorus* samples. We calibrated the phylogeny using three ancient samples from the well-dated site of Obłazowa WE in Poland^21^, which we assigned an age of 15 ka BP. The resulting phylogeny revealed two main lineages corresponding to *C. sungorus* and *C. campbelli*, which diverged approximately 85 ka BP (95% HPD: 140–49 ka BP; Figure 2). The ancient samples, tip-dated to between 32.5 and 9.6 ka BP, formed three highly supported clades that were sister to all modern *C. sungorus* individuals. The divergence times of these clades were estimated to range between 44 and 21 ka BP, whereas all modern *C. sungorus* samples coalesced around 16 ka BP. Notably, the temporal spans of the Late Pleistocene clades based on the estimated sample ages did not overlap.

**Fig. 3:**
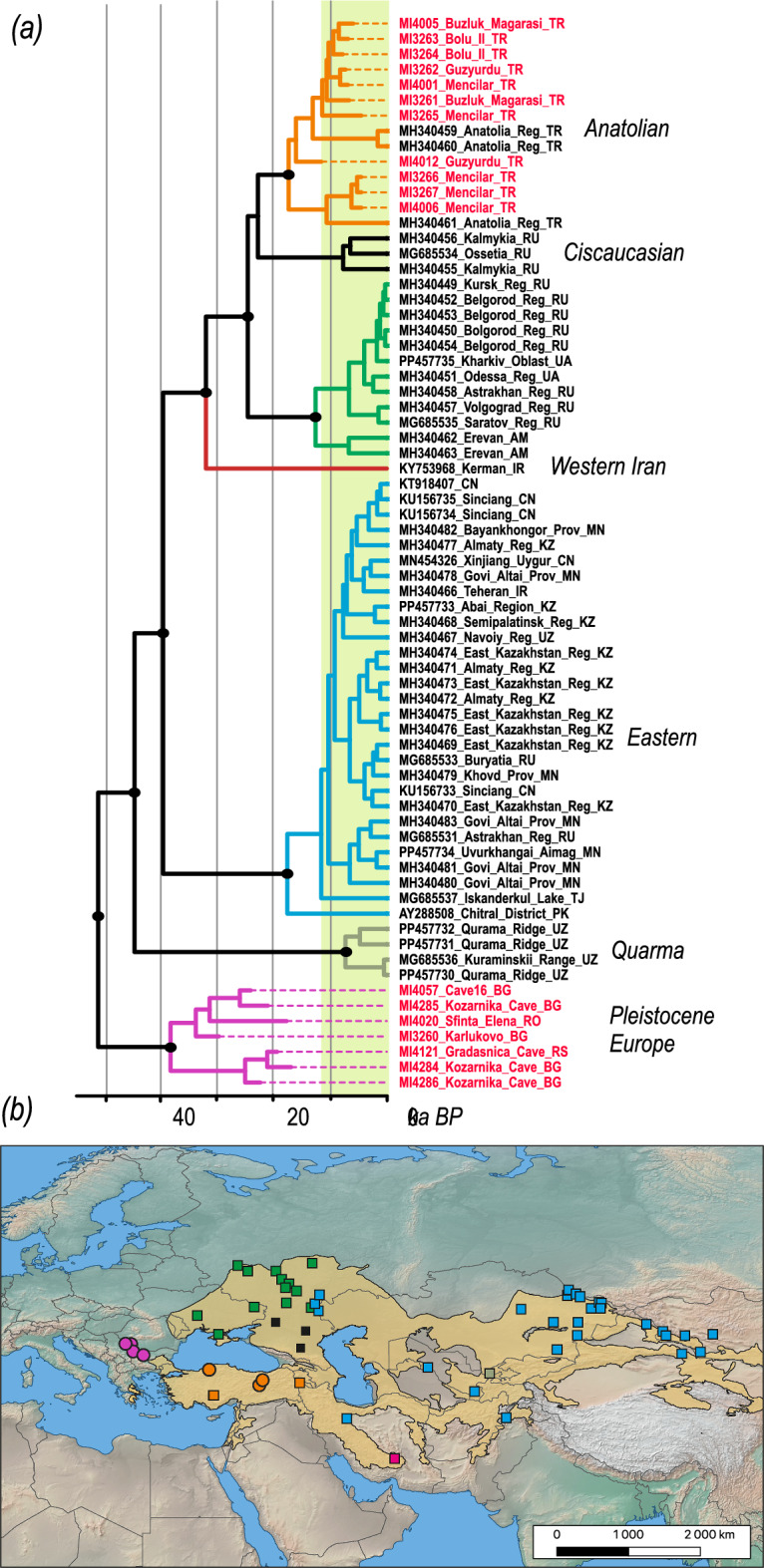
(**a**) Calibrated mitochondrial phylogeny of grey dwarf hamsters reconstructed in BEAST 1.10.5. Black dots at nodes indicate posterior probabilities >0.95. Tips are annotated with laboratory IDs, palaeontological sites and country codes. Tips of sequences from GenBank are annotated with accession numbers, sampling localities, and country codes. The green vertical band marks the Holocene. (**b**) Map showing sampling localities, coloured according to their phylogenetic placement. Circles represent samples newly sequenced in this study, while squares represent samples from GenBank. The yellow shaded area indicates the current distribution of the grey dwarf hamster based on the IUCN data^25^. The base map was downloaded from Natural Earth @naturalearthdata.com and modified using QGIS @qgis.org.

The grey dwarf hamster (*N. migratorius*) dataset was based on mtDNA cytochrome *b* (1140 bp) and comprised sequences of 73 *N. migratorius* specimens, including 18 ancient samples obtained in this study. None of the ancient samples came from sites securely dated enough to serve as calibration points. Instead, we applied mtDNA cytochrome *b* mutation rates previously estimated for field voles (*M. agrestis*)^22,23^. The resulting phylogeny revealed the existence of seven main lineages. The retrieved topology related to modern populations was similar to the previous reconstruction^24^, and all modern European samples were confined to three lineages: Anatolian, which included Late Holocene samples generated in this study; Ciscaucasian, which, positioned as a sister lineage to the Anatolian lineage, was weakly supported; and Western, including modern samples from Ukraine and Russia. The Late Pleistocene samples from the Balkans formed a distinct clade, which was sister to all modern lineages. The ages estimated for the ancient samples ranged between 29.7 ka BP and 16.9 ka BP. The divergence of the European Late Pleistocene lineage from the modern ones was relatively recent and estimated to be ~51 ka BP (95% HPD: 61.7–41.7 ka BP), while the divergence of the main lineages representing modern eastern, western, and Quarma populations was estimated to be between 44.5 and 39.5 ka BP (Figure 3).

## Discussion and conclusions

Species assignment of small hamsters based on tooth morphology is challenging because of their overall similarity. The presence of hairy-footed hamsters in Europe during the Late Pleistocene was first considered by Storch^26^ and was later robustly supported by numerous records from Sesselfelsgrotte (Lower Bavaria, Germany) based on the small size of the specimens^27^. However, Horáček and Lebedová^8^ recently challenged this hypothesis, showing that the teeth of hamsters from Sesselfelsgrotte fall well within the size variation of modern grey dwarf hamsters, and the size criterion alone cannot be used for species assignment. Consequently, despite certain doubts, they assigned the Central European Late Pleistocene small hamsters, in accordance with a monographic survey by Kowalski^7^, to *N. migratorius*. However, the doubts on relevance of traditionally applied discrimination criteria based on cranial phenotype traits and real taxonomic status of fossil representatives of the group started the present project with initial focus on the samples analysed by Horáček and Lebedová^8^ further supplemented with a number of records covering both fossil and extant ranges of the respective forms. In result, the ancient mitochondrial DNA analysis has clearly shown that both hairy-footed and grey dwarf hamsters were present in Late Pleistocene Europe. The geographic distribution of the two species, the hairy-footed hamster in Central and Western Europe and the grey dwarf hamster in the Balkans, is consistent with their ecological characteristics. At present, hairy-footed hamsters occupy higher latitudes than grey dwarf hamsters, up to 55°N and 45°N, respectively^12^. Hairy-footed hamsters are cold-steppe specialists, better adapted to low temperatures, and resilient to large temperature amplitudes. It is also worth mentioning that the Late Pleistocene Central European samples of *C. sungorus* exceed the range of dental measurement variation observed in extant populations, both in mean and maximum values^8^. This suggests an increase in body size within this clade under glacial conditions, a pattern that has been reported for various arvicolid species^28^.

The appearance of *C. sungorus* in Central Europe was associated with a faunal complex composed not only of dominant elements of glacial communities, such as *Stenocranius anglicus*, but also of demanding taxa of steppe habitats, including *Marmota*, *Hystrix vinogradovi*, *Allactaga*, *Lagurus*, *Lemmus lemmus*, *Spermophilus citelloides*, *Ochotona*, and *Spermophilus superciliosus*, among others^8^. These species can be considered as the index fossils of the mammoth steppe, a highly productive glacial zonal biome that spread during Marine Isotope Stage (MIS) 3 over the entire Palearctic region. *C. sungorus* was also recorded after the Last Glacial Maximum stage, and its last appearance in Central Europe is dated to the earliest Holocene (11.2 ka BP in Skalice, 10.8 ka BP in Martinka; Supplementary Table S1), similar to the last appearance data of other elements of the above-mentioned faunal complex. A similar pattern was observed for glacial populations of *N. migratorius* in the southern Balkans. Their expansion is thought to have been synchronous with the spread of *Lagurus lagurus* and *Eolagurus luteus*^29^. The boundary between the glacial ranges of these two Cricetinae species likely followed the Danube River, a region that also yielded the southernmost records of *Stenocranius anglicus*, including sites such as Sfânta Elena, Kozarnika, Razishka, Karlukovo, and Cave 16.

The essential aspect of this study was the estimation of calibrated phylogenies for both hamster species. In the absence of radiocarbon-dated specimens, calibration relied on indirect proxies; therefore, a critical evaluation of the resulting phylogenies is necessary to assess the robustness of the conclusions.

The calibration of the hairy-footed hamster mitochondrial phylogeny relied on three sequences obtained from specimens whose ages were assigned based on their stratigraphic position. Although the use of stratigraphy carries an inherent risk of temporal mismatch between specimens and sediment, particularly due to post-depositional disturbance, the Obłazowa WE site represents an exceptional case in this regard. The deposit comprises only three layers (I–III). The specimens originated from layer III which, together with layer II, is notably homogeneous. Their stratigraphic integrity is supported by an extensive set of at least 19 published radiocarbon dates derived from both large mammals, such as horse (*Equus ferus*) and reindeer (*Rangifer tarandus*) and small mammals, such as hare (*Lepus cf. timidus*), common vole (*M. arvalis*), root vole (*Alexandromys oeconomus*), and narrow-headed vole (*S. anglicus*)^11,21,30,31^. The calibrated median ages for these specimens consistently fell between 14 and 16 ka cal BP, with only a single charcoal-derived date yielding a slightly younger median age of 13.9 ka cal BP. Given this tight chronological clustering across taxa, it is highly unlikely that hairy-footed hamster samples differ substantially in age from the surrounding faunal assemblage.

For the grey dwarf hamster, a mitochondrial DNA mutation rate estimated from a different species was used. Previously, divergence times estimated for narrow-headed voles from a cytochrome *b* dataset using the same mutation rate (4×10⁻⁷ substitutions per nucleotide per year)^10^ yielded results very similar to those obtained from mitochondrial genome reconstructions calibrated with directly radiocarbon-dated samples^11^, supporting the validity of this substitution rate.

An additional measure of calibration accuracy is the consistency between the estimated ages of subfossil samples and their expected ages based on stratigraphic information (Supplementary Text S1). In the case of the grey dwarf hamster, three samples from layer 4b of Kozarnika Cave yielded molecular estimates of ages ranging from 22.3 to 16.9 ka BP (95% HPD), which are somewhat younger than the available conventional radiocarbon date for this layer (GifLSM-10677: 26,120 ± 100^32^; 95.4% range: 30,746–30,096 cal BP; calibrated here using OxCal 4.4^33^ and IntCal20 calibration curve^34^). A sample from the Gradašnica Cave was dated to 19.2 ka BP. Although no direct radiocarbon dates are available for this site, the age of two *Microtus arvalis* specimens from the same cave was previously estimated to be 27.0 and 21.5 ka BP^31^, placing the hamster sample plausibly within that timeframe. Only the age of specimen MI4057 from Cave 16, estimated to 24.0 ka BP, seems to be substantially underestimated. It originates from layer 8 which lies below the Campanian Ignimbrite tephra, suggesting a minimal age of ca. 40 ka.

Comparative data are available for the Siberian hairy-footed hamster from several sites. At Langmahdhalde, two radiocarbon dates from layer GH 8 range between 16,094–15,941 cal BP and 17,025–16,680 cal BP^35^, while a hamster from the same layer yielded a molecularly estimated age of 12.9 ka BP. At Skalice A/7, no radiocarbon dates were available, but the ages of three narrow-headed vole samples from layer 8 were estimated to be 17.1, 16.9, and 16.6 ka BP^11^. A hamster from this layer (MI4002) was slightly younger at 11.2 ka BP.

At Bišilu (Czechia), two hamster samples (MI4015 and MI4016) from layer 6 were estimated to be 25.1 and 26.4 ka BP, respectively. A single radiocarbon date for the same layer suggests a younger age (16,185–15,767 cal BP), but three narrow-headed vole samples from this layer previously yielded molecular age estimates of 35.5, 34.0, and 25.5 ka BP^11^, indicating that it may span a relatively long period. Only the oldest hamster samples appeared to be significantly underestimated in terms of age. Sample MI4442 from layer GH 15 at Hohle Fels (Germany) yielded an estimated age of ~33 ka BP, while the layer was tentatively assigned to MIS 5^16,36^. Similarly, sample MI3277 from Chlum 7B (Czechia) was estimated to be 30.5 ka BP, although the site is considered to represent an MIS 4/5 sequence^28,37^.

The overall good correspondence between the estimated specimen ages and the stratigraphic information suggests that the inferred coalescence times of the mtDNA lineages are unlikely to be substantially biased and can therefore be used to support the conclusions.

In both hamster species, the sequences of Late Pleistocene European samples fell outside the mitochondrial genetic diversity of the modern populations. However, the divergence of the clades formed by these sequences is relatively recent, occurring within the Last Glacial Period (115–11.7 ka BP), in contrast to European narrow-headed voles, whose divergence from Asiatic populations significantly predates the Eemian interglacial period.

The topology of both phylogenies, particularly that of hairy-footed hamsters, resembles the mitochondrial phylogeny of collared lemmings. In that species, Late Pleistocene populations formed a series of five temporally non-overlapping lineages, each encompassing samples from across much of the European continent and Asia^7,38,39^. This pattern was interpreted as an effect of serial population extinctions and recolonisations from the eastern Asiatic source populations^7,38^ however, recently, Lord et al.^39^ suggested that this may also be an effect of range contraction and survival of the refugial populations within Europe. The Eurasiatic diversity of the Late Pleistocene collared lemmings coalesced around 100 ka BP, placing the MIS 5 interglacial as the uppermost boundary for the expansion of European populations^39^.

In this study, we analysed mitochondrial DNA from Late Pleistocene and Holocene small hamster remains and demonstrated, contrary to expectations, that Europe was inhabited by two species: the hairy-footed hamster (*C. sungorus*) in Central and Western Europe and the grey dwarf hamster (*N. migratorius*) in the Balkans. The spread of the former appears to be associated with the mammoth-steppe conditions of MIS 3, whereas its last occurrence in Central Europe dates to the early Holocene. The topologies of mitochondrial phylogenies for both species support a scenario of repeated population expansions rather than long-term population continuity in Europe, as has been shown for the narrow-headed vole, which occupies a similar ecological niche.

## Materials and methods

### DNA Extraction, library preparation

#### Ancient samples

Fifty-nine molar teeth excavated from sites in Eastern and Central Europe, morphologically classified as small hamsters (subfamily Cricetinae), were examined (Supplementary Table S1).

The DNA extraction and pre-PCR library preparation procedure were carried out in a clean room type laboratory at the University of Warsaw’s Laboratory of Paleogenetics and Conservation Genetics. All precautions were taken to minimise the risk of contamination of samples with exogenous material and cross-contamination. No contemporary materials have been handled in this laboratory. After each stage of the work, the room was exposed to UV irradiation. Each molar was decontaminated with 0.5% sodium hypochlorite, rinsed twice with ultrapure water, and then crushed and lysed according to a protocol optimised for the recovery of very short DNA fragments^40^. To monitor possible contamination, a negative control containing no biological material from the isolation stage to sequencing was included. A half-USER treatment^41^ was used for most ancient samples to minimise the impact of DNA damage on downstream analyses (see Table S1). Single-stranded double-indexed libraries were constructed according to the protocol of Gansauge et al.^42^. The number of cycles for indexing PCR was estimated by qPCR, and the amplification reaction was performed using AccuPrime™ Pfx DNA Polymerase (Invitrogen).

#### Modern samples

Modern samples were processed in a dedicated laboratory physically separated from the clean laboratory. Three *C. sungorus* bone fragments originated from a collection at Charles University, originally acquired in the 1970 s from animals that had died naturally in private breeding. Genomic DNA was extracted using the DNeasy Blood & Tissue Kit (Qiagen) according to the manufacturer’s recommendations, then fragmented with a Covaris S220 sonicator and converted into a double-indexed double-stranded library following the protocol of Meyer and Kircher^43^, with modifications described by Baca et al.^10^. For each sample, three indexing PCRs were set independently for 15 cycles using AmpliTaq Gold™ MasterMix (Applied Biosystems™).

### Enrichment and sequencing

In-solution target enrichment of mitochondrial DNA (mtDNA) was performed using a myBaits Custom 20–40K probe set (Daicel Arbor Biosciences, USA) designed for Euarchontoglires^44^. Each hybridisation reaction was carried out simultaneously for up to 5 samples. Hybridisation was carried out according to a high-sensitivity protocol at 60ºC with minor modifications regarding the number of cycles for post-capture PCR (20 cycles instead of 10). For a subset of samples, custom bait made from the amplified mitogenome of *C. sungorus* was used. For bait preparation, we obtained a tissue fragment originating from a dyed individual of *C. sungorus* from the Animal Lab of Nicolaus Copernicus University in Toruń. DNA was isolated from the muscle tissue using the DNeasy Blood & Tissue Kit (Qiagen). The hybridisation reaction was performed twice at 65ºC for 22–24h. Post-capture PCR was performed in triplicate after each hybridisation using Herculase II Fusion Polymerase (Agilent) for 15 cycles. Enriched libraries were pooled and sequenced on the Illumina NovaSeq6000 platform using the 2 × 50 bp mode for ancient samples and on NextSeq550 using the 2 × 75 bp mode for modern samples at the NGS Core Facility at the Centre of New Technologies, University of Warsaw (CeNT UW).

### Sequencing reads processing

Raw reads were demultiplexed using bcl2fastq (Illumina). Adapters and low-quality nucleotides were trimmed, and overlapping reads were collapsed using AdapterRemoval v.2.3.1^45^. The filtered reads were mapped to *C. sungorus* (GenBank accession number: MH166880) and *N. migratorius* (NC_031802) mtDNA sequences using *bwa aln*^46^ and parameters optimised for ancient DNA (−o 2 −n 0.01 −l 1024). Both mtDNA references listed above were merged with the human mitochondrial reference sequence to filter out possible contaminants^47^. Only reads longer than 30 bp and with MapQ >25 were retained. Supposed PCR duplicates were removed using a custom script, samremovedup.py (https://github.com/pontussk/samremovedup). For ancient samples, two nucleotides were clipped from each end of the DNA molecules using the trimBam command from the BamUtil suite^48^. Consensus sequences were called using a custom script based on bcftools v.1.9 mpileup and ivar call^49^. We called only positions with coverage higher than 2 and if a base was supported by more than 75% of the reads. Initial species assignment was performed by comparing the mapping statistics to different mtDNA references. Alignment files were visually verified using Tablet^50^. The DNA damage pattern and size distribution were assessed using MapDamage 2^51^.

### Phylogenetic analyses

To determine the phylogenetic position of the samples, we reconstructed the mitogenomic phylogeny of Cricetinae species. The sequences were aligned using MAFFT v7.407^52^, and the final length of the mtDNA alignment was 15276 bp. The dataset included 16 modern mitogenomes and 33 ancient samples. Sequences from *Stenocranius gregalis* (MN199170) and *Chionomys nivalis* (NC_065750) were used as outgroups. To improve the overall efficiency of phylogenetic reconstruction, the alignment was processed using the BMGE v.1.12_1 software^53^. Maximum Likelihood phylogeny was reconstructed using IQ-Tree2^54^. The best-fitting substitution model was selected using ModelFinder (TIM2+F+I+R3), and branch support was assessed using an ultrafast bootstrap.

Based on their phylogenetic position, ancient samples were assigned to two species, and further phylogenetic reconstructions were performed separately for hairy-footed hamster (*C. sungorus)* and grey dwarf hamster (*N. migratorius)* datasets employing a Bayesian approach implemented in BEAST 1.10.5^55^. The best partitioning scheme and corresponding substitution models for each dataset were selected using PartitionFinder v.2^56^ (Supplementary Table S2).

The hairy-footed hamster (*C. sungorus*) dataset comprised 2230 bp mtDNA sequences encompassing cytochrome *b*, control region, tRNA-Thr, and tRNA-Pro and included sequences of modern *C. sungorus* (n=25), *Cricetiscus campbelli* (n=18) selected from GenBank, and all our ancient and modern *C. sungorus* samples. Three ancient samples came from a very well-dated site, Obłazowa WE, in Poland^21^. We assigned them an age of 15 thousand years BP and used them to calibrate the molecular clock. We used the Bayesian evaluation of temporal signal (BETS)^57^ to determine whether the dataset was suitable for estimating evolutionary rates. We used Marginal Likelihood Estimation (MLE) with Generalized Stepping Stone sampling^58^ to compare four models: two with ages assigned to samples with either a constant or uncorrelated relaxed clock versus two models with no information on sample ages (isochronous analyses) and two clock models. The model with sample ages assigned to sequences and a strict molecular clock was highly supported among the tested models, suggesting that our dataset is suitable for estimating the divergence time and ages of undated samples (Supplementary Table S3). Furthermore, we tested two tree priors, and as a result, we used a strict molecular clock and coalescent Bayesian SkyGrid tree prior (Supplementary Table S3). Next, we dated each sample separately using a normal prior (μ=25,000, σ=13,000, truncated at 0 years), and finally, we ran a joint analysis with all the sequences. In the joint analysis, we set a normal prior on the ages of the undated specimens which matched the posterior distributions of ages from the individual analyses (Supplementary Table S1). All BEAST analyses were run in duplicate and consisted of 50 million iterations sampled every 5 thousand generations.

The grey dwarf hamster (*N. migratorius*) dataset was based on mtDNA cytochrome *b* (1140 bp) and comprised sequences of 73 *N. migratorius* specimens, including 18 ancient samples obtained in this study. We used the MLE to test the tree prior (Supplementary Table S3). In result we used a strict molecular clock and a coalescent Bayesian SkyGrid tree prior. We used a cytochrome b mutation rate of 4E−7 substitutions/site year^−1^, previously estimated for field voles (*M. agrestis*)^22,23^. We set normal priors on the ages of the ancient samples based on available stratigraphic information (Supplementary Table S1). Two independent runs of 20 million generations sampled every 2 thousand generations were conducted.

Chain convergence and effective sample sizes were evaluated using Tracer v. 1.7^59^. The trees were summarised in TreeAnnotator with the first 10% of iterations discarded as burn-in.

## Supplementary Information

Supplementary Information 1.

Supplementary Information 2.

## Data Availability

Raw sequence reads and reads mapped to mitochondrial references have been deposited in the European Nucleotide Archive under project accession PRJEB89836. Consensus mtDNA sequences are available in GenBank under the accessions PV660946–PV660963 and PV684632–PV684649. The mtDNA alignments and BEAST XML files used for phylogenetic reconstruction have been deposited in the RepOD repository (https://doi.org/10.18150/EEVJR6).

## References

[1] Ratajczak, U., Stefaniak, K., Żeromska, A., Gagat, P. & Mackiewicz, P. Temporal and spatial differentiation of Pleistocene and recent *Saiga* deduced from morphometric analyses of cranial remains. *Hystrix* **32**, 18–26. 10.4404/hystrix-00338-2020 (2021).

[2] Nadachowski, A., Lipecki, G., Ratajczak, U., Stefaniak, K. & Wojtal, P. Dispersal events of saiga antelope (*Saiga tatarica*) in Central Europe in response to the climatic fluctuations in MIS 2 and the early part of MIS 1. *Quat. Int.* **420**, 357–362. 10.1016/j.quaint.2015.11.068 (2016).

[3] Taron, U. H. et al. ncient DNA from the Asiatic Wild Dog (*Cuon alpinus*) from Europe. *Genes* **12**, 1–19. 10.3390/genes12020144 (2021). 10.3390/genes12020144 PMC7911384 33499169

[4] Baca, M. et al. The Tien Shan Vole (*Microtus ilaeus*; Rodentia: Cricetidae) as a new species in the Late Pleistocene of Europe. *Ecol. Evol.* **11**, 16113–16125. 10.1002/ece3.8289 (2021). 34824815 10.1002/ece3.8289 PMC8601874

[5] Kowalski, K. Pleistocene rodents of Europe. *Folia Quat.* **72**, 3–389 (2001).

[6] Rabiniak, E. et al. Late Pleistocene and Holocene pikas (Mammalia, Lagomorpha) from Europe and the validity of *Ochotona spelaea*: New insights based on mtDNA analysis. *Palaeontol. Electron*10.26879/1241 (2023).

[7] Palkopoulou, E. et al. Synchronous genetic turnovers across Western Eurasia in Late Pleistocene collared lemmings. *Glob. Chang. Biol.* **22**, 1710–1721. 10.1111/gcb.13214 (2016). 26919067 10.1111/gcb.13214

[8] Horáček, I. & Lebedová, K. Cricetinae in the Quaternary fossil record of the Czech Republic and Slovakia (Rodentia: Cricetidae). *Lynx, n. s.* **53**, 365–415. 10.37520/lynx.2022.024 (2022).

[9] Sommer, R. S. Late Pleistocene and Holocene History of Mammals in Europe In *Handbook of the Mammals of Europe* (eds Hackländer, K. & Zachos, F. E.) 1–16 (Springer International Publishing) 10.1007/978-3-319-65038-8_3-1 (2020).

[10] Baca, M. et al. Highly divergent lineage of narrow-headed vole from the Late Pleistocene Europe. *Sci. Rep.* **9**, 17799. 10.1038/s41598-019-53937-1 (2019). 31780683 10.1038/s41598-019-53937-1 PMC6882798

[11] Baca, M. et al. Ancient DNA of narrow-headed vole reveal common features of the Late Pleistocene population dynamics in cold-adapted small mammals. *Proc. R. Soc. B-Biol. Sci.* **290**, 20222238. 10.1098/rspb.2022.2238 (2023). 10.1098/rspb.2022.2238 PMC9928523 36787794

[12] Kryštufek, B., Schenbrot, G. True Hamsters (Cricetinae) of the Palaearctic Region. (University of Maribor, University Press) 10.18690/um.fnm.1.2025 (2025).

[13] López-García, J. M. et al. Small-mammals from the Middle Pleistocene layers of the Sima del Elefante (Sierra de Atapuerca, Burgos, northwestern Spain). *Geologica Acta* **9**, 29–43. 10.1344/105.000001644 (2011).

[14] Bescós, G. C. Análisis filogenético de *Allocricetus* del Pleistoceno (Cricetidae, Rodentia, Mammalia). Allocricetus (*Cricetidae, Rodentia, Mammalia*) *from the Pleistocene*. *A phylogenetical approach. Coloquios de Paleontología* **1**, 95–113 (2003).

[15] Hir, J. *Cricetulus migratorius* (PALLAS 1773) (Rodentia, Mammalia) population from the Toros Mountains (Turkey) (With a special reference to the relation of *Cricetulus* and *Allocricetus* genera)*. *Folia Hist. Nat. Mus. Matraensis* **18**, 17–34 (1993).

[16] Luzi, E., Blanco-Lapaz, Á., Rhodes, S. E. & Conard, N. J. Paleoclimatic and paleoenvironmental reconstructions based on the small vertebrates from the Middle Paleolithic of Hohle Fels Cave, SW Germany. *Archaeol. Anthropol. Sci.* **14** (6), 107. 10.1007/s12520-022-01568-5 (2022).

[17] Nadachowski, A. Origin and history of the present rodent fauna in Poland based on fossil evidence. *Acta Theriol.* **34**, 37–53. 10.4098/at.arch.89.2 (1989).

[18] Neumann, K. et al. Molecular phylogeny of the Cricetinae subfamily based on the mitochondrial cytochrome *b* and 12S rRNA genes and the nuclear vWF gene. *Mol. Phylogenet. Evol.* **39**, 135–148. 10.1016/j.ympev.2006.01.010 (2006). 16483801 10.1016/j.ympev.2006.01.010

[19] Pan, X. et al. Phylogenomic analyses of hamsters (Cricetinae) inferred from GBS data and mitochondrial genomes. *Mol. Evol. Phylogenet.* **202**, 10.1016/j.ympev.2024.108241 (2025). 10.1016/j.ympev.2024.108241 39547600

[20] Lebedev, V. S. et al. Molecular phylogenetics and taxonomy of dwarf hamsters *Cricetulus* Milne-Edwards, 1867 (Cricetidae, Rodentia): Description of a new genus and reinstatement of another. *Zootaxa* **4387**, 331–349. 10.11646/zootaxa.4387.2.5 (2018). 29689907 10.11646/zootaxa.4387.2.5

[21] Lemanik, A. et al. The impact of major warming at 14.7 ka on environmental changes and activity of Final Palaeolithic hunters at a local scale (Orawa-Nowy Targ Basin, Western Carpathians, Poland). *Archaeol. Anthropol. Sci.* **12**, 66. 10.1007/s12520-020-01020-6 (2020).

[22] Herman, J. S., Searle, J. B. Post-glacial partitioning of mitochondrial genetic variation in the field vole. *Proc. R. Soc. B-Biol. Sci.* **278**, 3601–3607; 10.1098/rspb.2011.0321 (2011). 10.1098/rspb.2011.0321 PMC3189369 21508032

[23] Herman, J. S. et al. Land-bridge calibration of molecular clocks and the post-glacial colonization of scandinavia by the eurasian field vole *Microtus agrestis*. *PLoS One* **9**, e103949. 10.1371/journal.pone.0103949 (2014). 25111840 10.1371/journal.pone.0103949 PMC4128820

[24] Lebedev, V., Poplavskaya, N., Bannikova, A., Ryurikov, G. & Surov, A. Genetic differentiation in *Cricetulus migratorius* Pallas, 1773 (Rodentia, Cricetidae). *Mamm. Biol.* **92**, 115–119. 10.1016/j.mambio.2018.05.001 (2018).

[25] Kryštufek, B., Bukhnikashvili, A., Sozen, M., Isfendiyaroglu, S. *Nothocricetulus migratorius. The IUCN Red List of threatened species* https://www.iucnredlist.org/species/5528/115073390 (2016).

[26] Storch, G. Zur Pleistozän-Holozän-Grenze in der Kleinsäugerfauna Süddeutschlands Einleitung. *Z. Säugetierkunde* **39**, 89–97 (1974).

[27] van Kolfschoten, T. The smaller mammals from the Late Pleistocene sequence of the Sesselfelsgrotte (Neussig, Lower Bavaria) In *Sesselfelsgrotte VI. Natuwissenschaftliche* Untersuchungen. Wirbeltiere I. (eds Frend, G. & Reisch, L.) 27–118 (Franz Steiner Verlag, Stuttgart, 2014).

[28] Horáček, I. & Sánchez-Marco, A. Comments on the Weichselian small mammal assemblages in Czechoslovakia and their stratigraphical interpretation. *Neues Jahrb. Geol. Paläontol. Mh.* **9**, 560–576 (1984).

[29] Popov, V. Pliocene-Quaternary small mammals (Eulipotyphla, Chiroptera, Lagomorpha, Rodentia) in Bulgaria: biostratigraphy, paleoecology and evolution. In *The Pleistocene, Geography, Geology and Fauna* (eds Huard, G. & Gareau, J.) 109–235 (Nova Science Publishers, Inc., 2018).

[30] Żeromska, A. et al. Reconstruction of phylogeographic relationships and evolution of the tundra vole, *Alexandromys oeconomus* (Rodentia, Cricetidae), based on ancient DNA. *Zool. J. Linn Soc.* **205** (4), zlaf154. 10.1093/zoolinnean/zlaf154 (2025).

[31] Baca, M. et al. Ancient DNA reveals interstadials as a driver of common vole population dynamics during the last glacial period. *J. Biogeogr.* **50**, 183–196. 10.1111/jbi.14521 (2023).

[32] Guadelli, J. -L. et al*.* Une sequence du Paleolithique inferieur au Paleolithique recent dans les Balkans : La grotte Kozarnika a Orechets (nord-ouest De La Bulgarie) In *Les premiers peuplements en Europe. British Archaeological Reports International Series* 87–103 (Archaeopress, Oxford, 2005).

[33] Bronk, R. C. Bayesian analysis of radiocarbon dates. *Radiocarbon* **51**, 337–360. 10.1017/S0033822200033865 (2009).

[34] Reimer, P. J. et al. The IntCal20 Northern hemisphere radiocarbon age calibration curve (0–55 cal kBP). *Radiocarbon* **62** (4), 725–757. 10.1017/rdc.2020.41 (2020).

[35] Schürch, B., Wong, G. L., Luzi, E. & Conard, N. J. Evidence for an earlier Magdalenian presence in the Lone Valley of southwest Germany. *J. Archaeol. Sci. Rep.* **57**, 104632. 10.1016/j.jasrep.2024.104632 (2024).

[36] Conard, N. J. et al. The cultural and chronostratigraphic context of a new leaf point from Hohle Fels Cave in the Ach Valley of southwestern Germany. *Mitteilungen der Gesellschaft für Urgeschichte* **30**, 41–66. 10.51315/mgfu.2021.30003 (2022).

[37] Horáček, I. et al. Speleologie a výzkum kvartéru na Chlumu u Srbska: historie a současný stav (Speleology and Quaternary research at Chlum Hill near Srbsko: history and current achievements). *Český Kras* **42**, 5–22 (2016).

[38] Brace, S. et al. Serial population extinctions in a small mammal indicate Late Pleistocene ecosystem instability. *PNAS* **109**, 20532–20536. 10.1073/pnas.1213322109 (2012). 23185018 10.1073/pnas.1213322109 PMC3528586

[39] Lord, E. et al. Population dynamics and demographic history of Eurasian collared lemmings. *BMC Ecol. Evol.* **22**, 126. 10.1186/s12862-022-02081-y (2022). 36329382 10.1186/s12862-022-02081-y PMC9632076

[40] Rohland, N., Glocke, I., Aximu-Petri, A. & Meyer, M. Extraction of highly degraded DNA from ancient bones, teeth and sediments for high-throughput sequencing. *Nat. Protoc.* **13**, 2447–2461. 10.1038/s41596-018-0050-5 (2018). 30323185 10.1038/s41596-018-0050-5

[41] Rohland, N., Harney, E., Mallick, S., Nordenfelt, S. & Reich, D. Partial uracil-DNA-glycosylase treatment for screening of ancient DNA. *Philos. Trans. R. Soc. B-Biol. Sci.* **370**, 20130624–20130624. 10.1098/rstb.2013.0624 (2014). 10.1098/rstb.2013.0624 PMC4275898 25487342

[42] Gansauge, M. T., Aximu-Petri, A., Nagel, S. & Meyer, M. Manual and automated preparation of single-stranded DNA libraries for the sequencing of DNA from ancient biological remains and other sources of highly degraded DNA. *Nat. Protoc.* **15**, 2279–2300. 10.1038/s41596-020-0338-0 (2020). 32612278 10.1038/s41596-020-0338-0

[43] Meyer, M. & Kircher, M. Illumina sequencing library preparation for highly multiplexed target capture and sequencing. *Cold Spring Harb. Protoc.* **5**, t5448. 10.1101/pdb.prot5448 (2010). 10.1101/pdb.prot5448 20516186

[44] Saha, A. et al. The first complete mitochondrial genome data of the Afghan pika *Ochotona rufescens* (Lagomorpha, Ochotonidae), near the type locality. *Data Brief* **53**, 110246. 10.1016/j.dib.2024.110246 (2024). 38533117 10.1016/j.dib.2024.110246 PMC10964060

[45] Schubert, M., Lindgreen, S. & Orlando, L. AdapterRemoval v2: rapid adapter trimming, identification, and read merging. *BMC Res. Notes* **9**, 1–7. 10.1186/s13104-016-1900-2 (2016). 26868221 10.1186/s13104-016-1900-2 PMC4751634

[46] Li, H. & Durbin, R. Fast and accurate short read alignment with Burrows-Wheeler transform. *Bioinformatics* **25**, 1754–1760. 10.1093/bioinformatics/btp324 (2009). 19451168 10.1093/bioinformatics/btp324 PMC2705234

[47] Feuerborn, T. R. et al. Competitive mapping allows for the identification and exclusion of human DNA contamination in ancient faunal genomic datasets. *BMC Genom.* **21**, 1–10. 10.1186/s12864-020-07229-y (2020). 10.1186/s12864-020-07229-y PMC7708127 33256612

[48] Jun, G., Wing, M. K., Abecasis, G. R. & Kang, H. M. An efficient and scalable analysis framework for variant extraction and refinement from population-scale DNA sequence data. *Genome Res.* **25**, 918–925. 10.1101/gr.176552.114 (2015). 25883319 10.1101/gr.176552.114 PMC4448687

[49] Grubaugh, N. D. et al. An amplicon-based sequencing framework for accurately measuring intrahost virus diversity using PrimalSeq and iVar. *Genome Biol.* **20**, 1–19. 10.1186/s13059-018-1618-7 (2019). 30621750 10.1186/s13059-018-1618-7 PMC6325816

[50] Milne, I. et al. Using Tablet for visual exploration of second-generation sequencing data. *Brief Bioinform.* **14**, 193–202. 10.1093/bib/bbs012 (2013). 22445902 10.1093/bib/bbs012

[51] Jónsson, H., Ginolhac, A., Schubert, M., Johnson, P. L. F. & Orlando, L. MapDamage2.0: fast approximate bayesian estimates of ancient DNA damage parameters. *Bioinformatics* **29** (13), 1682–1684. 10.1093/bioinformatics/btt193 (2013). 23613487 10.1093/bioinformatics/btt193 PMC3694634

[52] Katoh, K. & Standley, D. M. MAFFT multiple sequence alignment software version 7: Improvements in performance and usability. *Mol. Biol. Evol.* **30**, 772–780. 10.1093/molbev/mst010 (2013). 23329690 10.1093/molbev/mst010 PMC3603318

[53] Criscuolo, A. & Gribaldo, S. BMGE (Block mapping and gathering with entropy): A new software for selection of phylogenetic informative regions from multiple sequence alignments. *BMC Evol. Biol.* 10 (1), 10.1186/1471-2148-10-210 (2010). 10.1186/1471-2148-10-210 PMC3017758 20626897

[54] Minh, B. Q. et al. IQ-TREE 2: New models and efficient methods for phylogenetic inference in the genomic era. *Mol. Biol. Evol.* **37**, 1530–1534. 10.1093/molbev/msaa015 (2020). 32011700 10.1093/molbev/msaa015 PMC7182206

[55] Suchard, M. A. et al. Bayesian phylogenetic and phylodynamic data integration using BEAST 1.10. *Virus Evol.* **4**, 1–5. 10.1093/ve/vey016 (2018). 10.1093/ve/vey016 PMC6007674 29942656

[56] Lanfear, R., Frandsen, P. B., Wright, A. M., Senfeld, T. & Calcott, B. PartitionFinder 2: new methods for selecting partitioned models of evolution for molecular and morphological phylogenetic analyses. *Mol Biol Evol.* **34** (3), msw260. 10.1093/molbev/msw260 (2016). 28013191 10.1093/molbev/msw260

[57] Duchene, S. et al. Bayesian evaluation of temporal signal in measurably evolving populations. *Mol. Biol. Evol.* **37** (11), 3363–3379. 10.1093/molbev/msaa163 (2020). 32895707 10.1093/molbev/msaa163 PMC7454806

[58] Baele, G., Lemey, P. & Suchard, M. A. Genealogical working distributions for bayesian model testing with phylogenetic uncertainty. *Syst. Biol.* **65** (2), 250–264. 10.1093/sysbio/syv083 (2016). 26526428 10.1093/sysbio/syv083 PMC5009437

[59] Rambaut, A., Drummond, A. J., Xie, D., Baele, G. & Suchard, M. A. Posterior summarization in bayesian phylogenetics using tracer 1.7. *Syst. Biol.* **67** (5), 901–904. 10.1093/sysbio/syy032 (2018). 29718447 10.1093/sysbio/syy032 PMC6101584

