# Supplementary material for: Biogeographic history of the Late Pleistocene and Holocene European small hamsters (subfamily Cricetinae)
Source: Sci Rep. 2026 Jan 8;16:4145. doi: 10.1038/s41598-025-34298-4 (PMC12859059; doi:10.1038/s41598-025-34298-4)
Supplement: Supplementary file 1 — Supplementary Information 1. [file 41598_2025_34298_MOESM1_ESM.docx]

Supplementary Material to

**Biogeographic history of the Late Pleistocene and Holocene European small hamsters (subfamily Cricetinae).**

Barbara Bujalska^1^, Michał Golubiński^1^, Danijela Popović^1^, Claudio Berto^2^, Nicholas J Conard^3^, Anna Lemanik^4^, Elisa Luzi^5^, Zoran Marković^6^, Adam Nadachowski^4^, Vasil Popov^7^, Ivan Horáček^8^, Mateusz Baca^1^

^1^Centre of New Technologies, University of Warsaw, Warsaw, Poland 
^2^Faculty of Archeology, University of Warsaw, Warsaw, Poland 
^3^Department of Early Prehistory and Quaternary Ecology, University of Tübingen, Tübingen, Germany 
^4^Institute of Systematics and Evolution of Animals, Polish Academy of Sciences, Kraków, Poland 
^5^Institue for Archaeological Sciences, University of Tübingen, Tübingen, Germany 
^6^Natural History Museum, Belgrade, Serbia 
^7^Institute of Biodiversity and Ecosystem Research, Bulgarian Academy of Sciences, Sophia, Bulgaria 
^8^Department of Zoology, Charles University, Prague, Czechia

# Supplementary Figures


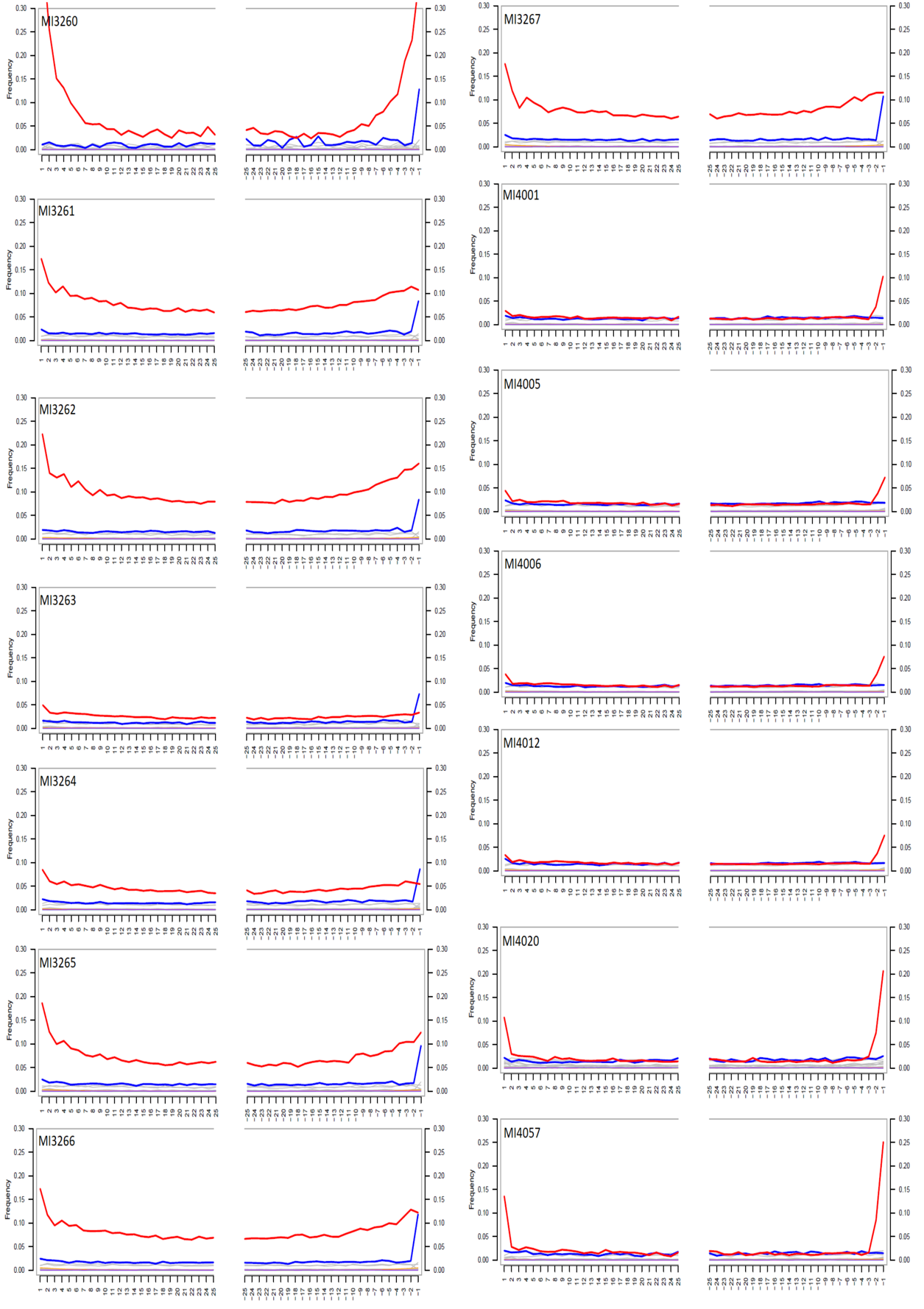


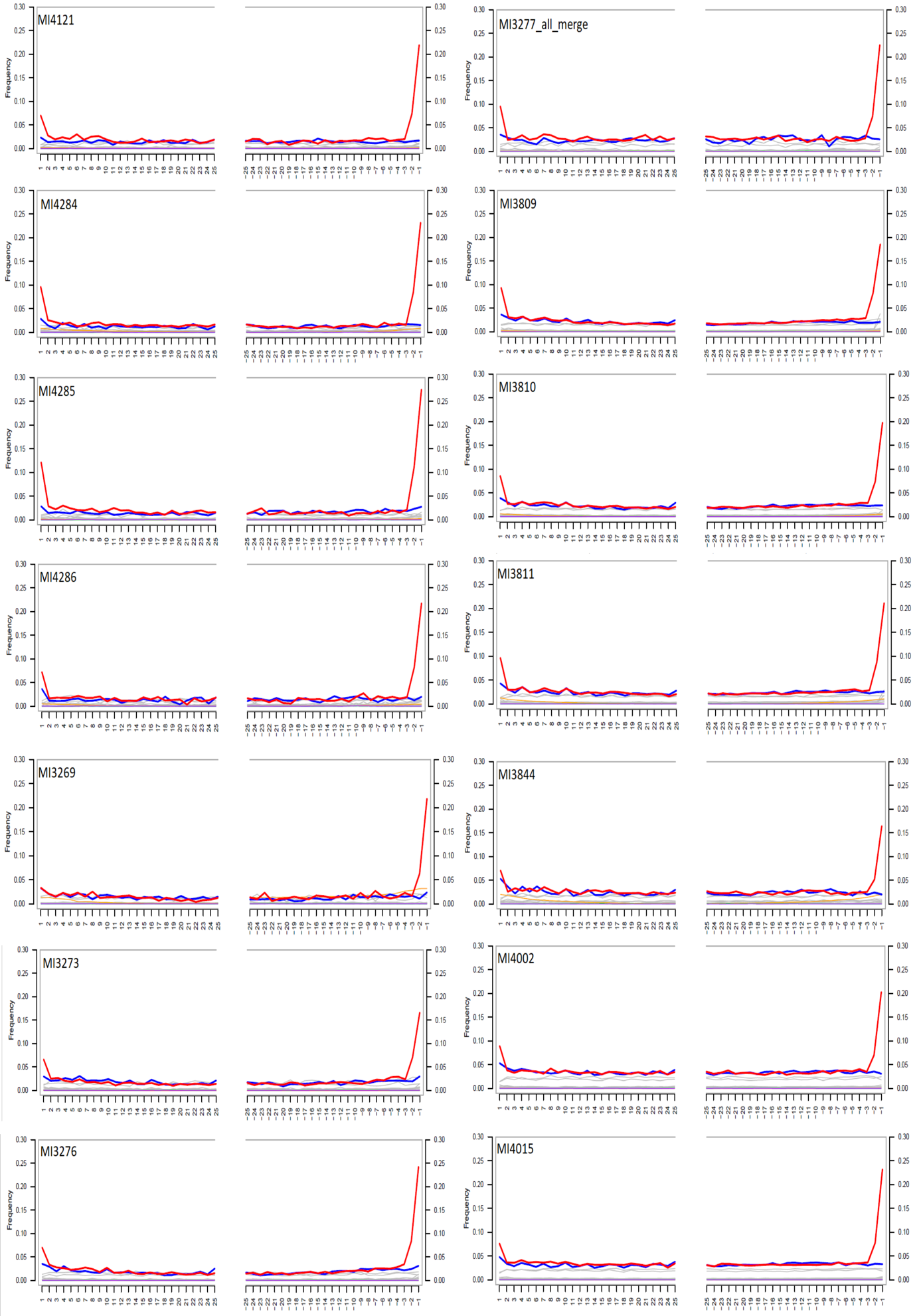


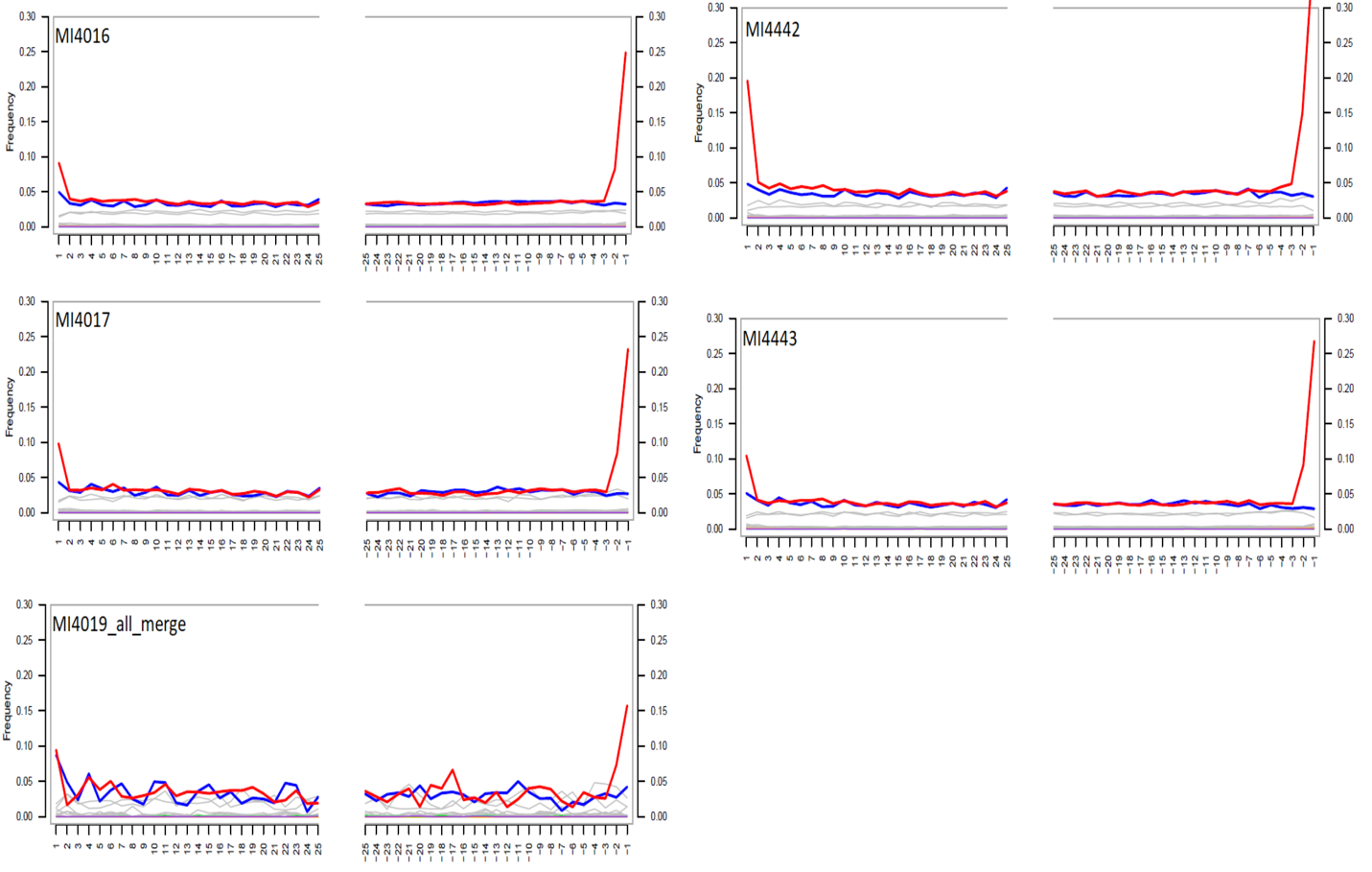


**
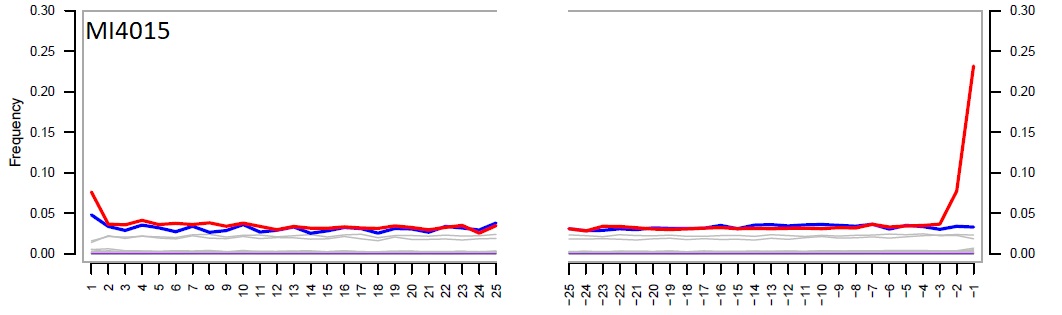

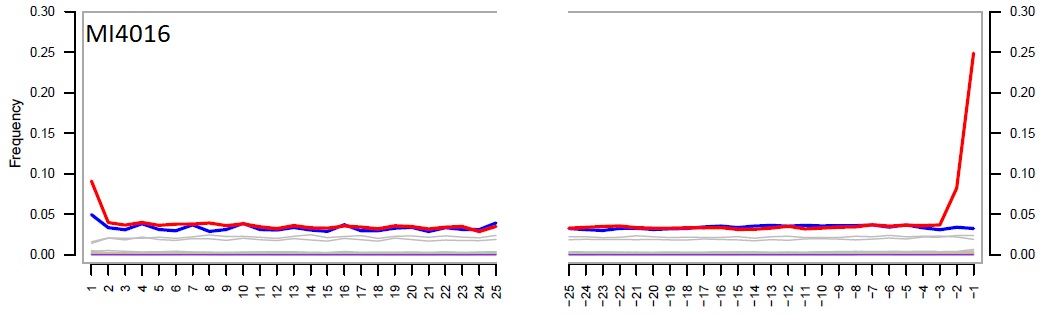

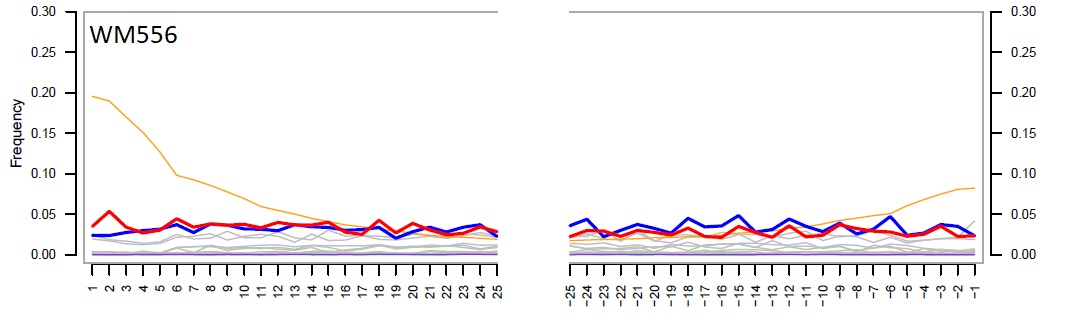

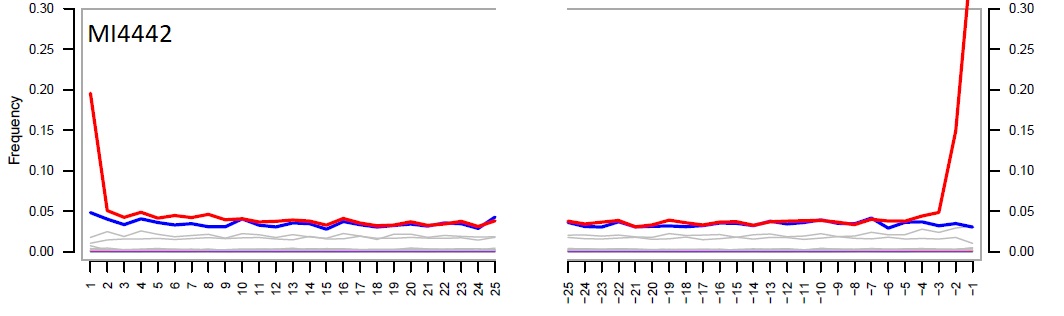

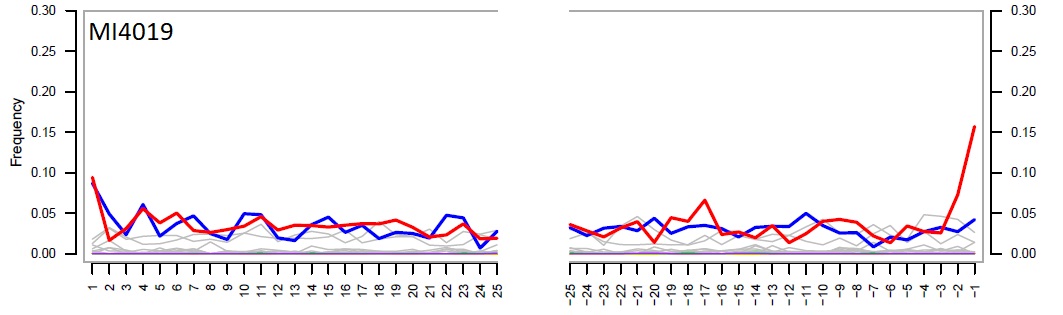

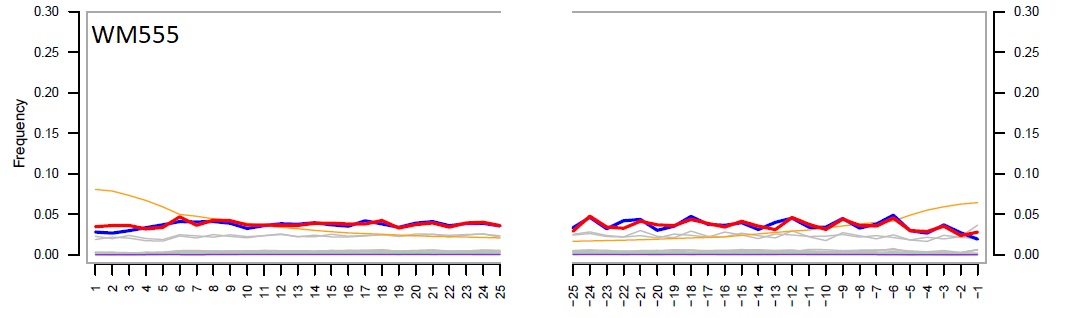

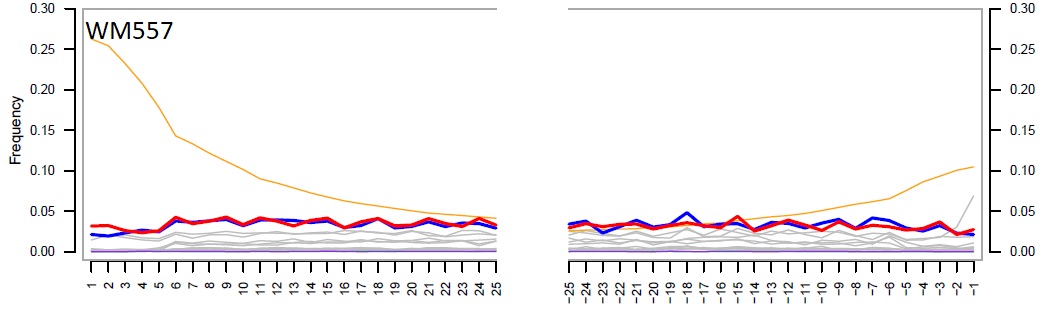
Figure S1.** Deamination profiles of the samples included in the phylogenetic analyses.


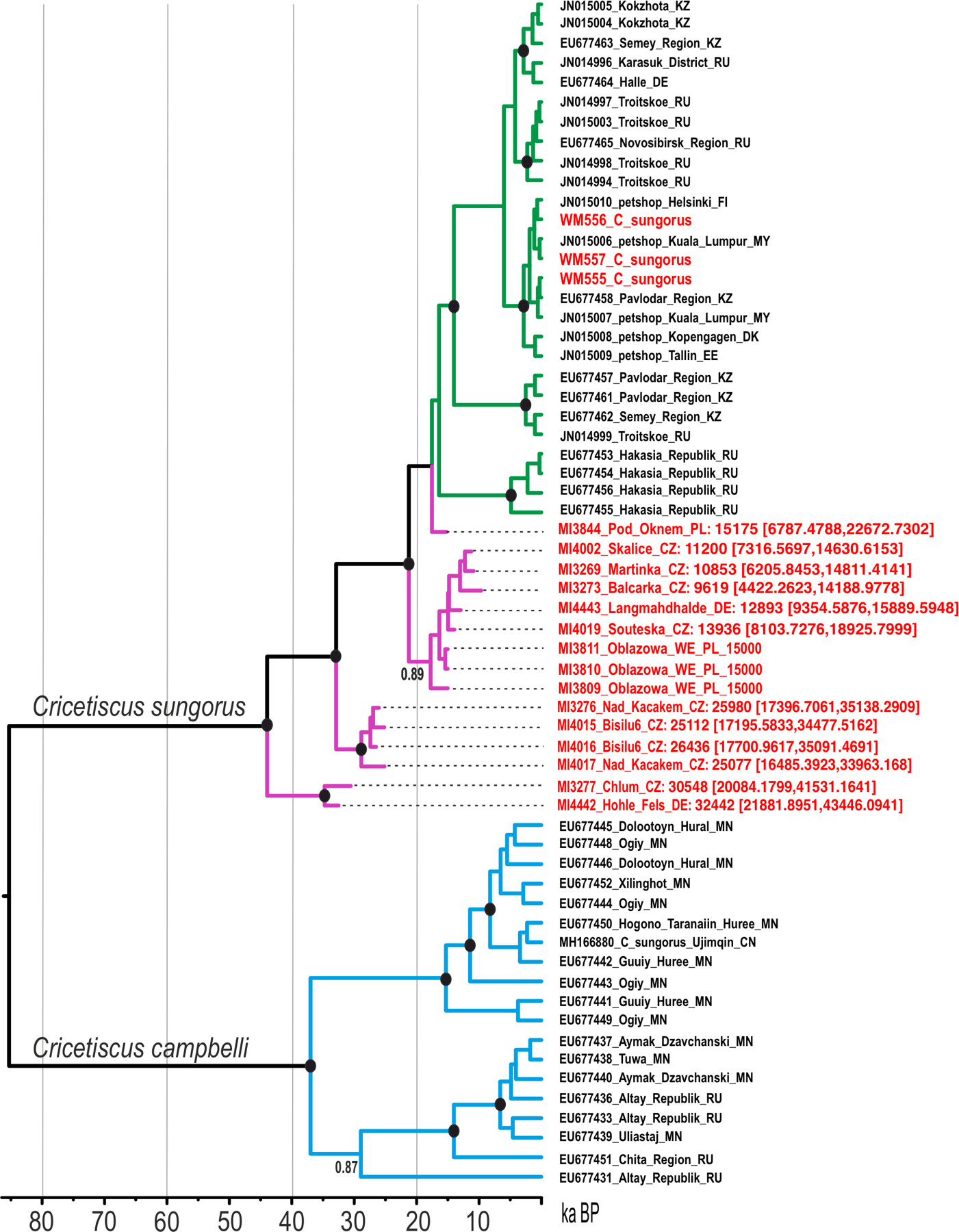


**Figure S2.** Calibrated phylogeny of hairy-footed hamsters (Cricetiscus sp.). Tips of molecularly dated samples are annotated with Lab ID, site, country code, median estimated age and age 95% HPD intervals.


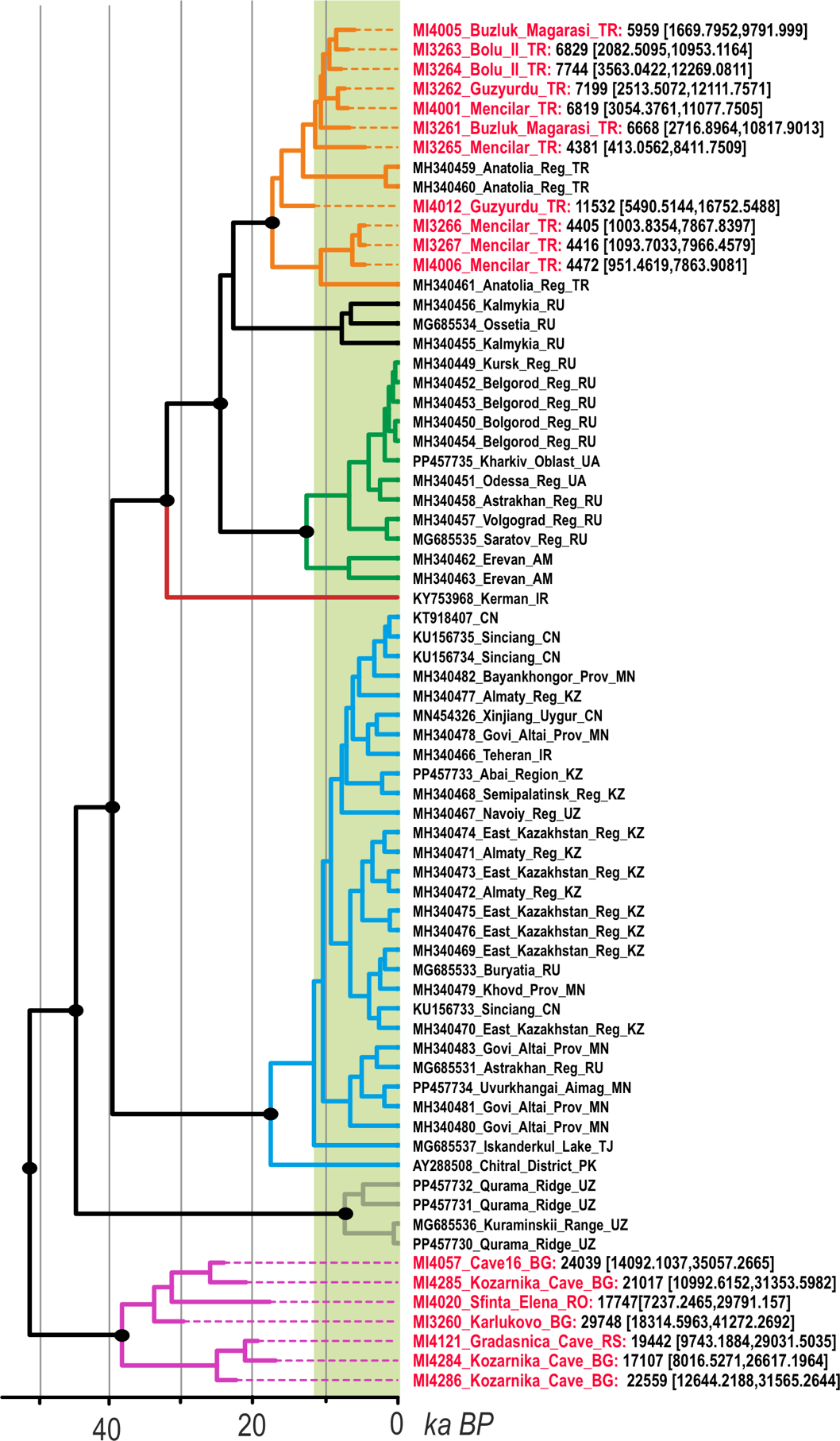


**Figure S3.** Calibrated mitochondrial phylogeny of grey dwarf hamsters. Tips of molecularly dated samples are annotated with Lab ID, site, country code, median estimated age and age 95% HPD intervals.

# Supplementary Materials and Methods

# Preparation of the enrichment bait

Based on the reference sequence from the Gene Bank (NC_042823), a set of six pairs of primers amplifying fragments of different lengths was designed to obtain the complete mitogenome sequence of *Cricetiscus sungorus*. Each fragment was amplified separately. The reaction mixture contained 20–50 ng of genomic DNA, 1× PrimeSTAR GXL buffer, dNTPs (200 μM each), 0.2 μM primers and 2.5 U PrimeSTAR GXL DNA polymerase, and the reaction was carried out in 50 μl. The reaction conditions were as follows: 35 cycles at 98 °C for 10 s, 60 °C for 15 s and 68 °C for 40 s. Amplicons were mixed equimolar and sonicated to fragments of about 200 bp using Covaris S220. The fragmented DNA was transformed into bait following the protocol described by Horn (2012).

Table 1. Primers used to amplify mitogenome of Cricetiscus sungorus.

| **Primer ID** | **Sequence (5’-3’)** | **Product length** |
| --- | --- | --- |
| Pho1_F | CCCAACGGACGCTATGAACT | 3.5 kb |
| Pho1_R | ATGTGAATGAGTGCGGGTGT |  |
| Pho2_F | TGTAGAATACGCCGCAGGTC | 3.8 kb |
| Pho2_R | TCGTAGAGATGGGAGGGCAA |  |
| Pho3_F | TCTGGCTTCTCCCTCCATCA | 3.9 kb |
| Pho3_R | CCGCATTCATATGGGCTTGC |  |
| Pho4_F | GACCCCCAACAGGCATTACA | 3.5 kb |
| Pho4_R | GCTGAGACTGGAGTTGGTCC |  |
| Pho5_F | TGGACGATCAGACGCAAACA | 3.7 kb |
| Pho5_R | TACCAAAGGCACGAGACCAC |  |
| Pho6_F | AGCCACACTCACACGATTCT | 3.6 kb |
| Pho6_R | TGGTAAACAGGCGGGATTCT |  |

# Description of paleontological sites

## Poland

**Obłazowa Cave** (western entrance), Western Carpathians, Nowa Biała

Obłazowa Cave (49°25’48”N, 20°09’36”E) is located in the south-western part of the Obłazowa Rock in the Pieniny Klippen Belt, Western Carpathians, Poland. The site yielded a number of important archaeological inventories, the oldest anatomically modern human (*Homo sapiens)* remains in Poland as well as abundant and diversified fauna discovered in a series of sediments divided into 21 layers [1–3]. The western entrance to the cave (Obłazowa Cave WE) was discovered in 2009 and intensively explored in years 2015–2016. A 2 x 2 m trench was situated next to the rocky wall directed to the west which forms an overhang. The sediments were located close to the limestone wall, under the overhang, and partly on the slope. Its depth reached ca. 60–70 cm. The profile consists of three layers, but remains of fauna was found only in layers II and III. At the top of layer II the Late Magdalenian inventories and fire place were discovered [4]. An accumulation of sediments took placed in the end of Late Pleistocene (GS-2a and GI-1e), ca. 16,1–14,0 ka cal BP, confirmed by numerous AMS radiocarbon dates [5,6].

**References**

1. Valde-Nowak, P., Nadachowski, A., Wolsan, M.. Upper Palaeolithic boomerang made of a mammoth tusk in south Poland. *Nature* **329**, 436–438 (1987).

2. Valde-Nowak, P., Nadachowski, A., Madeyska, T. Obłazowa Cave: human activity, stratigraphy and palaeoenvironment. 176 [38] (Institute of Archaeology and Ethnology Polish Academy of Sciences, 2003).

3. Valde-Nowak, P., Nadachowski, A. Micoquian assemblage and environmental conditions for the Neanderthals in Obłazowa Cave, Western Carpathians, Poland. *Quaternary International* **326–327**, 146–156;10.1016/j.quaint.2013.08.057 (2014).

4. Valde-Nowak, P., Kraszewska, A., Cieśla, M., Nadachowski, A. Late Magdalenian campsite in a rock shelter at the Obłazowa Rock in *Multas per Gentes et Multa per Saecula. Anici magistro et college suo ioanni Christopho Kozłowski dedicant* (ed. Valde-Nowak, P., Sobczyk, K., Nowak, M., Żrałka, J.) 175–183 (Institute of Archeology, Jagiellonian University in Krakow, 2018).

5. Nadachowski, A., Valde-Nowak, P. New Late Pleistocene faunal assemblages from Podhale Basin, Western Carpathians, Poland: preliminary results. *Acta Zool. Crac.* **58**, 181–194; 10.3409/azc.58_2.181(2015).

6. Lemanik, A. et al. The impact of major warming at 14.7 ka on environmental changes and activity of Final Palaeolithic hunters at a local scale (Orawa-Nowy Targ Basin, Western Carpathians, Poland). *Archaeol. Anthrop. Sci.* **12:** 66; 10.1007/s12520-020-01020-6 (2020).

**Pod Oknem Cave**

50°35’15’’N, 19°33’39’’E Pod Oknem Cave (also known as Jaskinia pod Bramą w Jastrzębniku) is located at an elevation of 370 m a.s.l. in the northern part of the Kraków-Częstochowa Upland, near the village of Kroczyce. The cave contains a well-preserved stratigraphic sequence spanning the Pleistocene and Holocene. Three fieldwork campaigns carried out between 2015 and 2022 allowed the investigation of three trenches: two inside the cave (trenches 1 and 2) and one outside (trench 3). Trench 2, situated within the cave, revealed a Late Pleistocene sedimentary sequence reaching up to 3 meters in thickness. Preliminary analyses on faunal and charcoal assemblages, along with sparse archaeological remains, suggest that this sequence accumulated between Marine Isotope Stage (MIS) 3 (layers 17 to 6) and MIS 2 (layers 5 and 3).

The small mammal assemblage from the lower layers (18 to 11) of Pod Oknem Cave shows a low minimum number of individuals (MNI), yet is characterized by high taxonomic diversity. The most abundant species include Alexandromys oeconomus, Clethrionomys glareolus, Dicrostonyx torquatus, Lemmus lemmus, Microtus agrestis, and Stenocranius anglicus. In the middle part of the sequence (layers 10 to 6), the assemblage is dominated by D. torquatus, S. anglicus, and L. lemmus, with overall lower diversity compared to the underlying layers.

A marked transition is observed between layers 6 and 5, where the assemblage becomes dominated by D. torquatus and S. anglicus, while L. lemmus and A. oeconomus disappear. Microtus arvalis and Ochotona sp. are present, though rare. Finally, layer 3 and 3A contains a more diverse assemblage.

The C. sungorus specimen consists of a right first upper molar (M1) recovered from the sandy layer 3–3A, located 40–50 cm below the surface of trench 2. No radiometric dates are available yet but preliminary analyses indicate that this layer was deposited during MIS 2. The associated small mammal assemblage is sparse, with a total minimum number of individuals (MNI) of 9, including Alexandromys oeconomus, Arvicola amphibius, C. glareolus, D. torquatus, L. lemmus, and M. agrestis. The occurrence of C. glareolus may be linked to Early Holocene disturbances, although this hypothesis is still under investigation.

## Czechia

**Bišilu cave**

49^0^ 57' N 14^0^ 6' E, 250 m a.s.l.

The site is located near Tetín, Beroun distr., central Bohemia. It is a cave entrance in a rocky cliff (250 m a.s.l.) completely infilled by sedimentary sequence of soil colluvia, limestone debris and loess loam at the base. Excavation revealed 16 different horizons (with total thickness > 3 m). The deeper part of the section (layers 4b–8 covering period from 11 ky cal. BP (layer 4b) to > 24 ky cal. BP (data for layer 7a) was particularly rich in fossils. In total, 28 species of small ground mammals were recorded with total MNI=602.

All late glacial and early Holocene layers (3b-8) show a diversified community of small mammals with dominant contribution of S. anglicus, Chionomys *nivalis, M. arvalis,* *A. oeconomus,* and a number of accessory taxa such as *Cricetus cricetus, C. sungorus* and *Allactaga major* with peak abundance in layer 6. The ^14^C aging suggested dates ranging from 11 ky cal. BP (layer 4) to > 24 ky cal. BP (layer 7a). The layer 6 was aged at 16 ky cal BP.

**Skalice**

49^0^ 54'N 14^0^ 6'E, 390 m a.s.l.

The cave is located near Měňany, Na Skalici cave, Beroun distr., central Bohemia. A stratigraphical section (>3 m of total thickness) in a sedimentary talus completely infilling a cave entrance in a rocky cliff close to local water spring. Horáček et al. [1] surveyed the results of the first stage of excavation in 1997. The most recent large-scale excavation was conducted in 2016–2017; analyses are in progress. Layer 7 (from which the items surveyed in this paper originate) represents a base loess with a glacial community dominated by *S. anglicus, M. arvalis/agrestis*, *A. oeconomus, C. nivalis*, *Dicrostonyx* sp. and *Ochotona pusilla*.

**References**

1. Horáček, I., Ložek, V., Svoboda, J., Šajnerová, A.: Environmental characters and karst occupancy in the late paleolitic and mesolithic colonisqtion of karst habitats (in Czech, English summary) in *Prehistorické jeskyně: katalogy, dokumenty, studie* (ed. Svoboda, J.) 313 – 354 (AÚ AV ČR Brno, 2002).

**Martinka**

48^0^ 53' N 16^0^ 31'E

A stratigraphic section situated in a rocky cliff of Pálava Mts. near Horní Věstonice, southerm Moravia. 10 well distinguished layers were samples covering the Vistulian pleniglacial - layers 9-10, late Pleistocene stage - layer 8, and preboreal - layer 7 stages. *C. sungorus* was recorded in layers 10 and 7 together with *S. anglicus, M. arvalis/agrestis, Dicrostonyx cf. gulielmi, A. oeconomus* or *Spermophilus citelloides*. Excavaions undertook in 1988 by V. Ložek and I. Horáček. For forther details see [1].

**References**

1. Horáček, I., Ložek, V. Biostratigraphic investigation of crevasse filling in the Martinka Cliff (in Czech with English summary). *Ceskoslovensky Kras* **41,** 83–99 (1990). –discussing also possible confusion on determination of small cricetins and possible occurence of *Phodopus* in central Europe

**Balcarka cave**

49°22’ N, 16°45' E

A large phreatic cave at bottom of spacious valley, situated in the centre of Moravian karst near a village Sloup, subjected to extensive excavations since 19th Century providing a very abundant bone material including almost all species of local fauna both Late Pleistocene and Holocene. The original stratigraphical setting of the sedimentary infill cannot be reconstructed. A rich remains of small vertebrtates recently (1988) collected by I. Horáček were obtained from talus under a chimney close to entrance corridor. It provided a typical Vistulian assemblage dominated by *L.*, *C. nivalis* with the sporadic presence of *D. torquatus*. Radiocarbon dating indicated for it the age of 33 200 cal BP.

**Nad Kačákem cave**

49^0^ 55'N 14^0^ 07'E, 250 m a.s.l.

Infill of a deep part of narrow subhorisontal tunnel with reddish-ochraceous loess-loam, supposedlsy of deluvial product of lower horizons of a thick entrance deposits completely excavated by Petrbok (1944). Most of currently analysed samples were collected by O. Fejfar and I. Horáček in 1982–1984. The samples revealed a rich fauna of the middle Vistulian (cal ^14^C 40.4–42.9 ky BP) composed of 38 vertebrate species with total MNI 142. Small mammals with 25 species and MNI 1371 dominated the sample. The most frequent species were *C. nivalis* (MNI 617), *S. anglicus* (MNI227) and *L. lemmus* (MNI 167), *C. sungorus* was represented by MNI 12.

**Chlum 7**

49^0^ 55'N 14^0^ 10'E, 310 m a.s.l.

Spacious chimney of the Srbské cave, a large cave system situated in Chlum hill near a village Srbsko, Bohemian karst, Central Bohemia. Loes-loam fillingh provided a rich material boith large mammals (*Crocuta, Panthera, Equus* etc.) and small mammals (17 spp., MNI 207) dominated by *M. arvalis, C. nivalis, Arvicola* sp. etc. Absence of *S anglicus* and low representation of *Dicrostonyx* indicates early Vistulian age of the assemblage. For further details see [1,2].

**References**

1. Horáček, I., Sánchez Marco, A. Comments on the Weichselian small mammal assemblages in Czechoslovakia and their stratigraphical interpretation. *N. Jb. Geol. Palaeont. Mh.* **9**, 560–576 (1984).

2. Horáček, I. et al.. Speleology and Quaternary research at Chlum Hill near Srbsko: history and current achievements (in Czech, English summary). Český Kras , **42**, 5–22 (2016).

**Soutěska I**

48^0^ 52'N 16^0^ 31'E

A stratigraphical section under a rocky slope of Soutěska gorge in Pálava Mts., southern Moravia, excavated 1979 by V. Ložek and resampled by I. Horáček in 1981. Besides few remains of small vertebrates a single specimen of *C. sungorus* was found in the bottom layer 7 supposedly of the late glacial age.

## Turkyie

**Buzluk Magarasi**

39^0^ 15'N 38^0^ 40'E

A stratigraphic section excavated in 1993 in the entrance corridor of the Buzluk cave near Harput (SE Turkey, Vilayet Elazig) by I. Horáček. Four layers (supposedly from early Holocene to Recent) provided in total remains of 21 species of small mammals (129 MNI) dominated by *Microtus irani, Apodemus arianus, Mesocricetus brandti, Meriones tristami* and *C. sugorus* (MNI 5).

**Guzyurdu**

39^0^ 58'N 39^0^ 37'E

A stratigraphic section denoted Guzyurdu 2A (from which the analysed samples come) was excavated in a rocky overhang near a village Guzyurdu (vilayet Bayburt) in 1995 by I. Horáček. The two layers distingushed in a section (both supposedly of the late Holocene age) provided remains of 19 spp. with MNI 67 including among other *Microtus obscurus, C. nivalis, Allactaga* cf*. williamsi, Mesocricetus* and 4 MNI *Nothocricetulus migratorius*.

**Bolu II**

40^0^ 30' N 31^0^ 25'E

Subfossil infil of a small cavern in a large traverine complex in vicinity of Bolu (NW Turkey, vilayet Bolu), supposedly remains of a *Bubo bubo* nesting place of the latest Holocene ages. The sample collected in 1990 by I. Horáček provided in total 277 MNI of 18 spp., dominated by *N. migratorius* (MNI 170) and *Microtus epiroticus* (MNI 60).

**Mencilar Magarasi**

41^0^ 30' N 32^0^ 40' E

A smaller cave in narrow valley western of Safranbolu (N Turkey, vilayet Karabuk) excavated in 1993 and 1994 by I. Horáček. In total it provided remains of 24 species (MNI 201) with dominant representation of *N. migratorius* (MNI 79) and *Mesocricetus* cf. *newtoni* (MNI 34), besides of *C. nivalis, M. epiroticus* etc.

## Romania

**Sfanta Elena**

44^0^ 32'N 21^0^ 70'E

A small cave situated in rocky environment close to top of a rignt bank of Danube river in the area of Iron Gate, SW Romania. The loes-like loam infill provided a small samples of vertebrate remains comprising of 44 individuals (MNI) of 11 species, including *L. anglicus* (one of the souther most records in Balkans), *O. pusilla*, *Mesocricetus newtoni, Mus macedonicus* etc. collected in 1991 by I. Horáček.

## Germany

**Hohle Fels**

48°22′45″ N, 9°45′20″ E

Hohle Fels Cave is located in the Ach Valley (SW Germany) and has been a focus of Paleolithic research since the 1870s. The archaeological excavations exposed a ca 6 m-thick sequence, spanning from the Middle Paleolithic to the Magdalenian. Geological Horizons (GH) 9 to 16 belong to the Middle Paleolithic. In 2020, the team recovered a leaf point at the top of GH 13, the first stratified find of its kind found in the region since the 1930’s [1].

The material analysed in this paper was originally assigned to *Allocricetus* vel *Cricetulus* sp., and belongs to GH 15. The micromammal assemblage for this GH includes *Neomys fodiens, Neomys milleri, Sorex* ex gr. *araneus, Sorex minutus, Sorex tundrensis, Talpa europaea; O. pusilla; A. oeconomus, Arvicola amphibius, C. nivalis, D. torquatus, L. lemmus, M. arvalis, S. anglicus, Sicista* cf. *subtilis,* and *Spermophiulus superciliosus* [2]*.*

There are no direct dates available for this GH 15, but the overlying stratum GH 12 yielded a ESR mean date of 62.5 +/- 4 ka BP [3]. Based on the comparison between GH 12 and 15, given the differences in the species composing the assemblages, the SDQ values for *A. amphibius* [4], and palaeoecological reconstructions, we assign GH 15 to the end of the Early Weichselian (MIS 5d-a) [2].

**Langmahdhalde**

48°34′1″ N, 10°12′47″ E

Langmahdhalde is a rock-shelter located in the Lone Valley (SW Germany). The excavation campaigns at the site took place between the year 2016-2024, under the direction of Prof. N. J. Conard (University of Tübingen).

The upper Geological Horizons (GH) 4-9 contain lithic tools, organic artefacts, in-situ hearths dating to the Magdalenian, while the lower GH 24-28 yielded evidences of a Middle Paleolithic occupation [5–7] .

The specimen analysed in this paper was originally assigned to *N. migratorius*, and originates from GH 8. The micromammal assemblage is still under study, and the list of species found at GH 8 so far includes: *Apodemus* sp., *M. arvalis, D. torquatus,* and *S. anglicus.*

Two radiocarbon dates for GH 8 yielded ages of 16,250 - 15,941 cal BP and 17,025 - 16,680 cal BP [8].

**References**

1. Rots, V., Coppe, J., Conard, N. J. A Leaf Point Documents Hunting with Spears in the Middle Paleolithic at Hohle Fels, Germany. *Mitteilungen der Gesellschaft für Urgeschichte* **2021**, 1–28; 10.51315/mgfu.2021.30004 (2021).

2. Luzi, E., Blanco-Lapaz, Á., Rhodes, S. E., Conard, N. J. Paleoclimatic and paleoenvironmental reconstructions based on the small vertebrates from the Middle Paleolithic of Hohle Fels Cave, SW Germany. *Archaeol. Anthropol. Sci.* **14**, 1–20 (2022).

3. Conard, N. J., Janas, A., Richard, M., Schürch, B., Tribolo, C. The cultural and chronostratigraphic context of a new leaf point from Hohle Fels Cave in the Ach Valley of southwestern Germany. *Mitteilungen der Gesellschaft für Urgeschichte* **30** (2021).

4. Heinrich, W.-D. Zur biometrischen Erfassung eines Evolutionstrends bei Arvicola (Rodentia, Mammalia) aus dem Pleistozän Thüringens. *Säugetierkundliche Informationen* **2**, 3–21 (1978).

5. Conard, N. J., Zeidi, M., Janas, A. Ausgrabungen an der Langmahdhalde im Lonetalerreichen mittelpaläolithische. *Schichten. Archaol. Ausgrabungen Baden-württemb.*, 53–57 (2023).

6. Conard, N. J., Janas, A., Luzi, E. Weitere Ausgrabungen an der Langmahdhalde im Lonetal und Erkenntnisse zu den Umweltverhältnissen während des Letzten Glazialen Maximums. *Archäologische Ausgrabungen Baden-württemb.* **2021** (2022).

7. Wong, G. L., Drucker, D. G., Starkovich, B. M., Conard, N. J. Latest Pleistocene paleoenvironmental reconstructions from the Swabian Jura, southwestern Germany: Evidence from stable isotope analysis and micromammal remains. *Palaeogeogr. Palaeoclimatol. Palaeoecol.* **540**, 109527; 10.1016/j.palaeo.2019.109527 (2020).

8. Schürch, B., Wong, G. L., Luzi, E., Conard, N. J. Evidence for an earlier Magdalenian presence in the Lone Valley of southwest Germany. *J. Archaeol. Sci. Reports* **57**, 104632; 10.1016/j.jasrep.2024.104632 (2024).

## Bulgaria

**Karlukovo cave 4**

A cave in a left bank of Iskar river ca 2 km N of a village Karlukovo (Stara Planina Mts., NW Bulgaria). Clayed sandy infill of a chimney provided a rich material of fossil remains composed of at least 696 individuals (MNI) of 31 mammalian and 3 non-mammalian vertebrate species (dominated with bats, namely *Pipistrellus pipistrellus*), incl. *M. newtoni, C. nivalis, Arvicola* sp., *Microtus* cf. *arvalis, Lagurus lagurus* etc. collected in 1978 by I. Horáček. For more details see [1].

**References**

1. Horáček, I.. Notes on the glacial-time environment in Northern Bulgaria (in Czech with English summary). *Ceskoslovensky Kras* **32**: 95-103 (1982).

**Cave 16**

43°10 37.31"N 24°4’20.33"E

Cave 16 is a large rock niche, half-filled with sediments, located in Praebalkan region, Karlukovo, north-western Bulgaria. At the entrance, the sediments were exposed by erosion, creating a well-stratified profile over 5 m thick. During pilot archaeological excavations in 1984–1993, the profile was cleared, and its stratigraphy was studied. Fifteen layers were identified [1,2]. At present and in the recent geological past, the cave is and has been a nesting place for many petrophilous birds, including owls. This is evidenced by numerous owl pellets on the modern surface of the sediments and the high accumulation of bones in the deposits from layer 15 upwards. Based on the ecological appearance of the stratigraphic small mammal assemblages, the layers formed two distinct groups, namely, upper (layers 9–1) and lower (layers 15–10). Among the layers forming the upper series, particular attention was paid to layer 7, which consists of volcanic ash. It resulted from one of the most powerful volcanic eruptions in southern Italy, known as the Campanian Ignimbrite, dating from ca. 40,000 years BP [3]. The assemblages from the lower part of the profile are characterised by a high proportion of forest and mesophilic species and thermophilic sub-Mediterranean elements; the inhabitants of the steppes have a low share. Quantitative paleoecological reconstructions [4,5] suggest that during the formation of the lower part of the sediment sequence (layers 14–10), the temperatures were similar to the modern ones. However, the humidity for most of this period was higher than the present humidity. The climate and vegetation were comparable to those in the mountains of Central Europe. The small mammal assemblages at the top of the profile show a high percentage of species characteristic of open landscapes. The layers of the upper part of the profile (layers 9-1) formed in a cooler climate, especially in the layers deposited before and after the volcanic ash. Winter temperatures were approximately 10 degrees lower than today. The seasonal temperature contrast was significant. The rainfall was less than today. In the age’s context of volcanic ash and based on the ecological appearance of small mammals and quantitative climatic reconstructions, layers 14–10, showing a milder climate, are most likely to be referred to the end of the last interglacial period; layers 10–8 reflect the progressive cooling during the early Last Glacial period (Marine Isotope Stage (MIS) 4–3), corresponding to the time interval between 90,000 and 50,000 years BP; layers 6–1 correlate with the Last Glacial Maximum (LGM).

**Kozarnika cave**

Kozarnika cave is situated near the village of Oreshets, in northwestern Bulgaria (22.702377° E, 43.651854° N). The stratigraphic information can be found in [6]. Analysis of the small mammal fossils indicates that the sequence of sediments discovered in the cave's entrance hall spans the last million years ([7. This timeline was subsequently validated through magnetostratigraphic studies ([8]. Based on the micromammals found and the changes observed in the evolutionary lines of several vole species, the sequence is divided into four biozones [7]: Kz-A1 (layers 3 to 9a), Kz-A2 (layers 9c to 10b), Kz-B1 (layer 11a), and Kz-B2 (layers 11b to 13). The first pair of biozones corresponds to the Early Pleistocene epoch (Upper Biharian). The studied samples (MI4284, MI4285, MI4286) originated from layer 4b, part of biozone Kz-A1. This biozone's layers suggest the presence of harsh climate conditions, evidenced by the formation of frost structures, along with a decline in the rate of deposition from wind-blown materials [6]. Layer 4b has an uncalibrated date of 26,120 +/- 100 years [9].

**References**

1. Popov, V. V.  Quaternary small mammals from deposits in Temnata – Prohodna Cave system in  *Temnata Cave. Excavations in Karlukovo Karst Area, Bulgaria* (ed. Ginter, B.,  Kozłowski, J. K., Laville, H.) 11–53 (Jagiellonian University Press, 1994).

2. Popov, V. V.  The small mammals (Mammalia: Insectivora, Chiroptera, Lagomorpha, Rodentia) from Cave 16 and the paleoenvironmental changes during the Late Pleistocene in   *Temnata cave. Excavations in Karlukovo Karst Area* (ed. Ginter, B.,  Kozłowski, J. K., Guadelli, J-L., Laville, H.) 159–240 (Jagiellonian University, 2000).

3. Giaccio, B. et al. The Campanian Ignimbrite and Codola tephra layers: two temporal/stratigraphic markers for the Early Upper Palaeolithic in southern Italy and eastern Europe. *J. Volcanol. Geotherm.* **177** (1), 208-226 (2008).

4. Popov, V. V., Di Canzio, E., Giaccio, B. Late Quaternary small mammals and paleotemperatures in Bulgaria and Italy. *Acta Zool. Bulg.* **66** (1), 89-108 (2014).

5. Popov, V. V.  Pliocene-Quaternary small mammals (Eulipotyphla, Chiroptera, Lagomorpha, Rodentia) in Bulgaria biostratigraphy, paleoecology, and evolution in *The Pleistocene* (ed*.*  Huard, G., Gareau, J.) 109–235 (Nova Science Publishers Inc, 2018).

6. Sirakov, N. et al. An ancient continuous human presence in the Balkans and the beginnings of human settlement in western Eurasia: A Lower Pleistocene example of the Lower Palaeolithic levels in Kozarnika cave (North-western Bulgaria). *Quaternary International*, **223-224**, 94–106 (2010).

7. Popov, V. V., Marinska, M. An almost one million year long (Early to Late Pleistocene) small mammal succession from the archaeological layers of the Kozarnika Cave in Northern Bulgaria. *Courier Forschungesinstitut Senckenberg* **257**, 79–92, Frankfurt a. M. (2007).

8. Muttoni, G. et al. An early Brunhes (<0.78 Ma) age for the Lower Paleolithic tool-bearing Kozarnika cave sediments, Bulgaria. *Quat. Sci. Rev.* **178**, 1–13; 10.1016/j.quascirev.2017.10.034 (2017).

9. Guadelli J.-L. et al... Une séquence du Paléolithique inferieur au Paléolithique récent dans les Balkans : La grotte Kozarnika à Oreshets (Nord-Ouest de la Bulgarie) in *Les Premiers Peuplements En Europe* (ed. Molines, N., Moncel, M. H., Monnier, J. L.). *BAR. IS.* **1364**, 87–103 (2005).

## Serbia

**Gradašnica cave**

N 44° 27' 45" E 22º 13' 42"

Gradašnica cave is located on the west side of Veliki Greben and Veliki Strnjak, nearby the Miroč village, above the Danube gorge. The cave’s monumental entrance is 35 m high and 15 m wide. Its altitude is approximately 380 m above the sea level. The main channel caved in Jurassic limestone is approximately 300 m long. The cave belongs to the spring-type of caves. First palaeontological investigation of this cave started in 1998. High water level throughout the year and the reduced accessibility to all parts (too low ceilings and too narrow passages) conditioned a limited choice of sediment collection sites. Approximately 50 kg of sediments were collected from the left ‘dead-end’ passage on the entrance of the cave. This passage, which is very narrow, contains a wider area of approximately 2.5 m^2^ where the sediments were neither disturbed by water nor by human activity. The porous clay sediment, which covers the limestone slope, was nowhere thicker than 15 cm. Variations in its lithological composition were undetectable, and layers were not recognised. After sieving the sediment, the remains of animals from different classes are sorted: Amphibia, Reptilia*,* Aves and Mammalia. The mammal remains were the most numerous, representing approximately 80% out of 2500 items: *Rhinolophus hipposideros, Rhinolophus ferrumequinum, Myotis myotis, Таlра europea, S. araneus, N. fodiens, Crocidura leucodon, Spermophilus citelus, M. newtoni, C. migratorius, C. cricetus, C. glareolus, A.* *amphibius, M. arvalis, Microtus subterraneus, C. nivаlis, Nannospalax leucodon, Glis glis, Muscardinus avelanarius, S. subtilis, Apodemus flavicollis/Аpodemus sylvaticus, Rattus* *rattus, O. pusilla, Lepus* sp., *Vulpes vulpes*, *Martes martes*, *Ursus* sp., *Bovidae* sp. The remains were accumulated in a place which is certainly not the habitat of any of these species. The bones did not bear any signs of being transported by water on any other geomorphological agents. Therefore, the most probable means of transporting and accumulation of this material is by the means of predators. Although no recent material was discovered, such a cave is a natural habitat for some of the owl species in the past and occasionally for some members of carnivora (*Mustela nivalis*, *V. vulpes* and *Martes* sp.). Analysis of the pattern of skull and mandible breakage showed that most remains were part of owl prey. Some of the species are extinct in the area at present (*O. pusilla*, *M. newtoni*, *C. migratorius*, *S. subtilis*), whereas the others are still present . The fossil *Rodentia* and *Lagomorpha* fauna from the Gradašnica cave sediments may be characterised by a mixture of steppe and forest faunal elements, with several species belonging to other biotopes, such as forest-steppe, mountain meadows, stony mountain peaks and wet forest clearings. None of the species found in the fossil material is extinct.
